# Phase 1 Safety and Immunogenicity Evaluation of ADVAX, a Multigenic, DNA-Based Clade C/B' HIV-1 Candidate Vaccine

**DOI:** 10.1371/journal.pone.0008617

**Published:** 2010-01-25

**Authors:** Sandhya Vasan, Sarah J. Schlesinger, Yaoxing Huang, Arlene Hurley, Angela Lombardo, Zhiwei Chen, Soe Than, Phumla Adesanya, Catherine Bunce, Mark Boaz, Rosanne Boyle, Eddy Sayeed, Lorna Clark, Daniel Dugin, Claudia Schmidt, Yang Song, Laura Seamons, Len Dally, Martin Ho, Carol Smith, Martin Markowitz, Josephine Cox, Dilbinder K. Gill, Jill Gilmour, Michael C. Keefer, Patricia Fast, David D. Ho

**Affiliations:** 1 Aaron Diamond AIDS Research Center, New York, New York, United States of America; 2 The Rockefeller University, New York, New York, United States of America; 3 International AIDS Vaccine Initiative, New York, New York, United States of America; 4 University of Rochester Medical Center, Rochester, New York, United States of America; 5 International AIDS Vaccine Initiative Core Laboratory, Imperial College, London, United Kingdom; 6 EMMES Corporation, Rockville, Maryland, United States of America; University of New South Wales, Australia

## Abstract

**Background:**

We conducted a Phase I dose escalation trial of ADVAX, a DNA-based candidate HIV-1 vaccine expressing Clade C/B' *env, gag, pol, nef*, and *tat* genes. Sequences were derived from a prevalent circulating recombinant form in Yunnan, China, an area of high HIV-1 incidence. The objective was to evaluate the safety and immunogenicity of ADVAX in human volunteers.

**Methodology/Principal Findings:**

ADVAX or placebo was administered intramuscularly at months 0, 1 and 3 to 45 healthy volunteers not at high risk for HIV-1. Three dosage levels [0.2 mg (low), 1.0 mg (mid), and 4.0 mg (high)] were tested. Twelve volunteers in each dosage group were assigned to receive ADVAX and three to receive placebo in a double-blind design. Subjects were followed for local and systemic reactogenicity, adverse events, and clinical laboratory parameters. Study follow up was 18 months. Humoral immunogenicity was evaluated by anti-gp120 binding ELISA. Cellular immunogenicity was assessed by a validated IFNγ ELISpot assay and intracellular cytokine staining. ADVAX was safe and well-tolerated, with no vaccine-related serious adverse events. Local and systemic reactogenicity events were reported by 64% and 42% of vaccine recipients, respectively. The majority of events were mild. The IFNγ ELISpot response rates to any HIV antigen were 0/9 (0%) in the placebo group, 3/12 (25%) in the low-dosage group, 4/12 (33%) in the mid-dosage group, and 2/12 (17%) in the high-dosage group. Overall, responses were generally transient and occurred to each gene product, although volunteers responded to single antigens only. Binding antibodies to gp120 were not detected in any volunteers, and HIV seroconversion did not occur.

**Conclusions/Significance:**

ADVAX delivered intramuscularly is safe, well-tolerated, and elicits modest but transient cellular immune responses.

**Trial Registration:**

Clinicaltrials.gov NCT00249106

## Introduction

With an estimated 33 million people living with HIV/AIDS globally, and roughly 2.5 million new infections in 2007 alone, the need for an efficacious vaccine to prevent or attenuate HIV-1 infection remains paramount [1]. In the People's Republic of China, an estimated 700,000 people are living with HIV/AIDS, in an epidemic spread both through sexual transmission and injection drug use. The prevalence of HIV infection among injection drug users in Yunnan province, which borders Myanmar, Laos, and Vietnam in the “golden triangle” region, has increased dramatically in the last ten years, to over 40% in several prefectures [2]. In a separate study, the annual incidence rate of new HIV infections among intravenous drug users in Guangxi province was found to be 3.1% [3].

The Aaron Diamond AIDS Research Center has pursued the development of a multigenic vaccine regimen based on the predominant clade C/B' circulating recombinant form of HIV-1 from Yunnan, China, CRF 007 [4]. After codon-optimization and certain safety mutations, matched sequences from the *env, gag, pol, nef*, and *tat* genes were inserted into both a naked DNA plasmid backbone (ADVAX) and a modified vaccinia ankara (MVA) viral vector (ADMVA) as described by Y. Huang et al. and Z. Chen et al., respectively [5], [6]. These vectors were initially chosen based on reports of improved cellular immunogenicity of DNA- and MVA-based vaccines when used in a prime-boost combination in humans with a variety of antigens [7]–[9] and on their ability to control viremia after multiple routes of simian human immunodeficiency virus (SHIV) challenge in rhesus macaques [10], [11].

The Phase I trial described in this report was designed to assess the safety, tolerability and humoral and cellular immunogenicty of ADVAX as a stand-alone HIV-1 vaccine candidate. A parallel Phase I study of the ADMVA vaccine alone was conducted separately, as reported in the accompanying manuscript.

## Methods

### Study Setting

The study was conducted at the Rockefeller University Hospital in New York City, USA, and the University of Rochester Medical Center in Rochester, New York, USA. The protocol for this trial and supporting CONSORT checklist are available as supporting information: see Checklist S1 and Protocol S1. This trial is registered at clinicaltrials.gov, registry number NCT00249106, http://clinicaltrials.gov/ct2/show/NCT00249106

### Participants

Healthy men and women aged 18–60 years were eligible for participation if they were not at high risk for HIV-1, as defined by having none of the following activities in the six months prior to enrollment: unprotected vaginal or anal sex with a known HIV-1-infected person or casual partner, injection drug use, acquisition of a sexually transmitted disease, or sex work for money or drugs. Participants agreed to safe sexual practices and effective contraception to avoid pregnancy throughout the duration of the 18-month study. Participants had to demonstrate a clear understanding of the possibility of HIV-1 seropositivity due to vaccine-induced antibodies in the event of a humoral immune response to encoded HIV-1 antigens. Exclusion criteria included chronic medical conditions, clinically significant abnormal laboratory parameters, infection with Hepatitis B or C virus, or recent receipt of a vaccine or blood transfusion.

### Ethical Complianc

This study was approved by the Institutional Review Boards of the Rockefeller University Hospital and the University of Rochester Medical Center. Individual participants in this study provided written informed consent after appropriate review, discussion and counseling by the clinical study team. The trial was monitored by the International AIDS Vaccine Initiative (IAVI). The study was conducted in compliance with International Conference on Harmonization - Good Clinical Practice (ICH-GCP).

### Interventions

The ADVAX vaccine is a 1∶1 mixture of two DNA plasmids derived from the pVax vector (Invitrogen™) containing clade C/B', codon-optimized HIV-1 gene sequences. The first plasmid expresses Env under the PCMV promoter and Gag under the human elongation factor 1α (PhEF1α) promoter, while the second expresses Pol under the PCMV promoter and a Nef-Tat fusion under the PhEF1α promoter as previously described [5]. ADVAX cGMP manufacturing, quality control testing and real-time stability studies were conducted at Vical Inc. (San Diego, CA).

This study was randomized, dose-escalating, and double blind with respect to active vaccine or placebo. Safety and tolerability of ADVAX or placebo in each dosage group were evaluated by an independent Data Safety Monitoring Board at least 14 days after the 12^th^ volunteer had received the second injection, and prior to initiation of enrollment of the next dosage group. The study design is summarized in Table 1. The 0.2 mg low dose was chosen based on safety considerations, to minimize the initial exposure of this novel investigational vaccine. The maximum dose of 4.0 mg was chosen based primarily on manufacturing constraints, as this was the maximum amount of ADVAX that could be concentrated into a 1.0 mL intramuscular injection volume.

**Table 1:** Study Design.

Group	Vaccine Dosage	Volunteers Receiving Vaccine∶Placebo	Vaccination Schedule (Months)	Total Follow Up (Months)
Low	0.2 mg	12∶3	0, 1, 3	18
Middle	1.0 mg	12∶3	0, 1, 3	18
High	4.0 mg	12∶3	0, 1, 3	18
**Total**		36∶9		

### Objectives

The primary objective was to evaluate the safety and tolerability of three intramuscular injections with ADVAX at three different dosage levels in healthy HIV-uninfected adults. The secondary objective was to evaluate the humoral and cellular immunogenicity of ADVAX versus placebo at each dosage.

### Outcomes

Primary endpoints were designed to evaluate the safety of ADVAX in human volunteers. Local reactogenicity (including pain, tenderness, erythema, edema, skin damage, induration, and formation of crust, scab or scar) and systemic reactogenicity (including fever, chills, headache, nausea, vomiting, malaise, myalgia, arthralgia, and rash) were assessed by telephone three days following vaccination and by history and physical examination two weeks after vaccination. Subjects were monitored for adverse events, general health and clinical laboratory parameters at each study visit.

Secondary endpoints were designed to evaluate the cellular and humoral immunogenicity of ADVAX. Blood for cellular immunogenicity analyses was collected at pre-vaccination baseline, two weeks after each vaccination, and at weeks 28, 36, 52, and 78 to follow longer term responses. Serum for humoral immunogenicity analyses was collected at pre-vaccination baseline, four weeks after each vaccination, and at weeks 28, 36, 52, and 78 to follow longer term responses. Cellular immunogenicity was assessed by IFNγ ELISpot on frozen peripheral blood mononuclear cells (PBMCs) at the IAVI Core laboratory at the Imperial College, London, as previously described [12]. Peptides for stimulation were synthesized by Anaspec (Freemont, CA) and pooled at the IAVI Core laboratory. Peptide pools consisted of 15mers overlapping by 11 amino acids matched to the Clade C/B' sequences encoded in the vaccine, and spanned all gene inserts.

For each pool, the ELISpot value was defined as the mean replicate (maximum 4) count minus the mean background count. Four criteria had to be fulfilled for an ELISpot value to be considered a positive response: 1) for each peptide pool, a single value had to be >99% of all pre-vaccination and placebo values for that pool, and >38 Spot Forming/10^6^ Cells (SFC) count; 2) the mean count had to be >4 times the mean background SFC count; 3) the mean background had to be <55 SFC/10^6^ PBMCs; and 4) the coefficient of variation had to be ≤70% across the replicate wells.

#### Cell stimulation

ELISpot-positive samples were tested for phenotype, cytokine secretion, and antigen-specific proliferation using polychromatic flow cytometry. Cryopreserved PBMCs were thawed rapidly at 37°C and rested overnight, then washed and resuspended in RPMI media with 10% v/v FCS. 8.7×10^5^ cells were co-incubated with 30 µg peptide pools or 20 µg SEB (Sigma-Aldrich, St. Louis, MO), CD107 PECy5 (Becton Dickinson, Franklin Lakes, NJ), Brefeldin A (Sigma-Aldrich, St. Louis, MO) and BD Golgistop (Becton Dickinson, Franklin Lakes, NJ) for 6 hours at 37°C, then at 4°C for no longer than 18 hours.

#### Staining and flow cytometry

Plates were washed twice in PBS by centrifugation, stained with 100 µL VIVID for 20 minutes, and then washed twice in FACSwash buffer (2% bovine serum albumin with 0.15% sodium azide). Cells were then surface stained with anti-CD4 QD605, anti-CD8 pacific orange (Invitrogen, Carlsbad, CA), anti-CD27 FITC (Beckton Dickinson, Franklin Lakes, NJ), and anti-CD45RO (Beckman Coulter, Fullerton, CA) for 20 minutes at room temperature, washed twice in PBS, then fixed and permeabilized by incubating in BD Cytofix Cytoperm solution for 20 minutes at 4°C. Cells were washed twice in BD Cytofix Cytoperm wash buffer and then stained intracellularly with anti-CD3 QD655 (Invitrogen, Carlsbad, CA), anti-IFN-γ PE Cy7, anti-MIP-1β PE, anti-TNF-α A700 and anti-IL-2 APC (Beckton Dickinson, Franklin Lakes, NJ). Cells were acquired on a custom-built BD LSR II cytometer. At least 500,000 events were collected. Data were analyzed using FlowJo (Treestar), PESTLE and SPICE (courtesy Mario Roederer, Vaccine Research Center) software.

#### Humoral immunogenicity

Binding antibodies to Clade C gp120 (NIH AIDS Reagent Program) were assessed by ELISA at pre-vaccination baseline and two weeks after each vaccination, as described by Huang et al. [13]. In parallel, anti-gp160, anti-p24, or anti-gp36 Group M/O antibodies were assessed using the Genetic Systems™ HIV-1| HIV-2 PLUS O EIA Kit (Bio-Rad Laboratories, Hercules, CA), at the New York State Department of Health. Those samples that were positive were further evaluated by the Genetic Systems™ HIV-1 Western Blot Kit (Bio-Rad Laboratories, Hercules, CA) and for viral load quantification using the Roche Amplicor HIV-1 Monitor v1.5 RNA-PCR Kit (Roche Diagnostic Systems, Indianapolis, IN) to differentiate a response to vaccine from *de novo* HIV infection. Results were monitored by an independent physician to maintain blinding of the clinical study team.

### Sample Size

Each of the three dosage groups consisted of 15 volunteers randomized in a 4∶1 ratio of vaccine to placebo (Table 1). The small sample size was chosen for an exploratory dose-escalation study of this novel product while investigating safety and tolerability of the vaccine. Based on a 10% event rate in the placebo group (n = 9), there was at least 80% power to detect a significantly greater event rate of 58% or more in the active group (n = 36) at level α = 0.05 using Fisher's exact one-sided test.

### Randomization and Blinding

The randomization schedule was prepared by the statisticians at the Data Coordinating Center at the EMMES Corporation, Inc. The randomization list was sent to Fisher Clinical Services for labeling and packaging of study vaccine and placebo in a double blinded fashion. Study site staff and volunteers remained blinded with respect to the allocation of placebo or vaccine, but not dosage group.

### Statistical Methods

Data from all participants, including those lost to follow up and those not completing the vaccination series, were included in the analyses. The rate of local and systemic reactogenicity events was used to assess the differences between dosage groups. Fisher's exact test was used for 2×2 tables, and the Cochran-Armitage trend test was used to investigate trends in event rates with increasing dosage.

## Results

### Participant Flow

**Figure 1:**
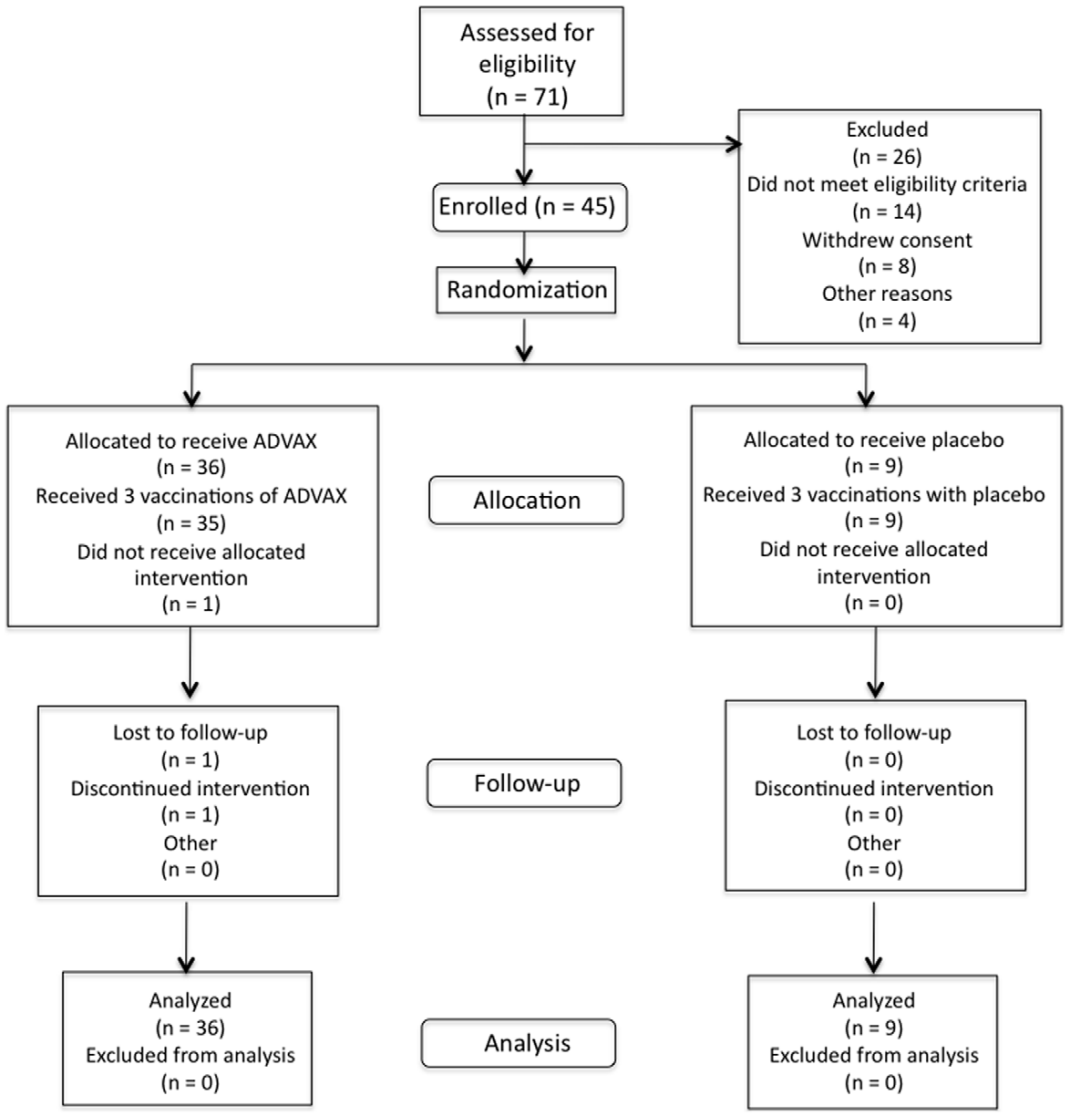
Clinical Trial Participant Flow Diagram.

As shown in Figure 1, 71 volunteers were screened for this study, of whom 45 were enrolled. The majority of the 26 screen failures were due to medical abnormalities: 7 due to chronic medical conditions, and 7 due to abnormalities on screening laboratories or urinalysis. Eight volunteers withdrew consent after completing the screening process. Of the remaining four screen failures, three were assessed by the study team as being unable to comply with the protocol, and one completed screening after the trial was fully enrolled. The average interval from date of screening to enrollment was 16 days, ranging from 3–35 days. All three vaccinations of either ADVAX or placebo were administered to all but one volunteer, who missed the final vaccine due to relocation. Two participants in total did not complete the trial for reasons unrelated to the vaccine or the study. One participant was lost to follow up due to relocation after receiving only two of the three scheduled vaccinations. The second participant, who had a prior history of depression, committed suicide seven weeks after completing her vaccination series, for reasons unrelated to vaccination.

### Recruitment

The low dosage group of volunteers was enrolled from December 2003 through January 2004, and followed until July 2005. The mid dosage group was enrolled from March 2004 through April 2004, and followed until September 2005. The high dosage group was enrolled from May 2004 through June 2004, and followed until November 2005. Baseline demographic and clinical characteristics for all trial participants are listed in Table 2.

**Table 2:** Baseline Demographics.

	ADVAX Low	ADVAX Mid	ADVAX High	Placebo	All Subjects
**Gender**					
Male	5	3	6	7	21
Female	7	9	6	2	24
**Age**					
Mean	34.5	34.6	31.8	35.7	34.0
Range	23–55	22–46	22–49	18–52	18–55
**Race/Ethnicity**					
Caucasian	10	9	10	8	37
Asian	0	1	1	0	2
African American	0	1	0	0	1
Hispanic or Latino	0	1	0	1	2
Native American or Alaskan Native	1	0	0	0	1
Native Hawaiian or Other Pacific Islander	1	0	1	0	2
Other/Unknown					

### Vaccine Reactogenicity and Adverse Events

**Figure 2:**
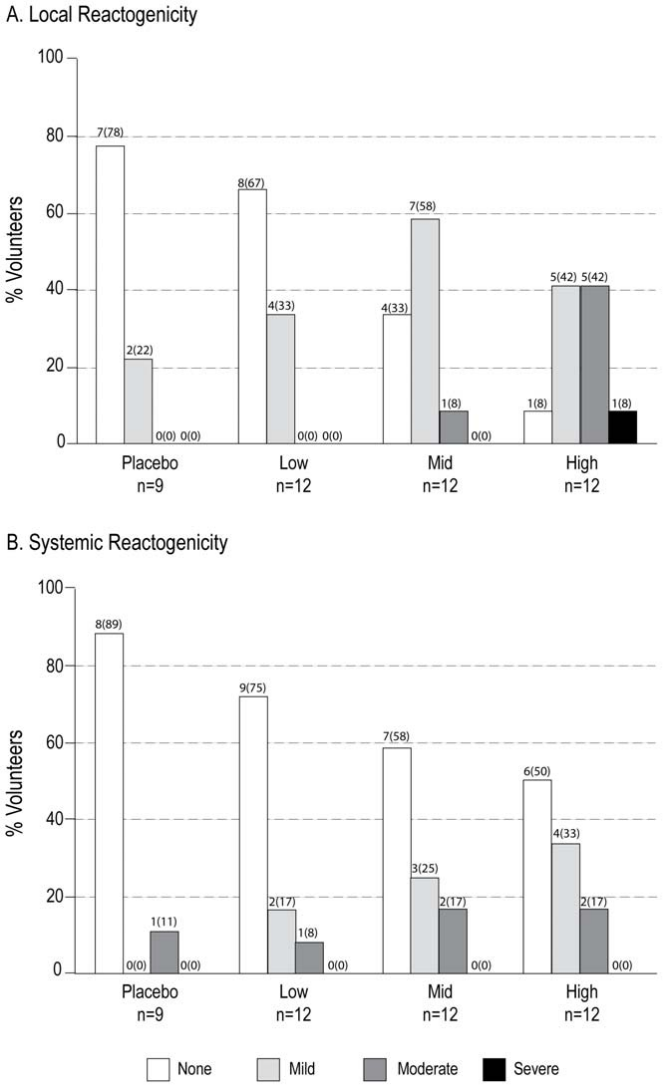
Local and Systemic Reactogenicity by Dosage Group. Panels A and B depict the percentage of volunteers experiencing local or systemic reactogenicity, respectively, by severity and dosage group. Total responses and (percentage of responses) are depicted above each bar. The proportion of volunteers experiencing moderate or severe local reactogenicity increased with increasing dosage (two-tailed Cochran-Armitage trend test, p = 0.0040). A similar comparison of systemic reactogenicity was not statistically significant (p = 0.738).

The percentage of volunteers experiencing local and systemic reactogenicity in each dosage group is presented in Figure 2. The most frequently reported local reactogenicity event in all dosage groups was pain and/or tenderness at the injection site, followed by mild erythema/skin discoloration. The most frequently reported systemic symptom was headache, followed by subjective fever. These local and systemic events were mostly mild and usually resolved prior to the subsequent visit (within 3–14 days). The proportion of volunteers experiencing moderate or severe local reactions increased significantly with increasing dosage (0%, 8% and 50% in the low, mid and high dose groups, respectively: two-tailed Cochran-Armitage trend test, p = 0.004), whereas dose had no significant effect on systemic symptoms (p = 0.738).

Only one volunteer experienced a serious adverse event, judged not related to vaccination (suicide in a volunteer with a history of depression). Of the 177 non-serious adverse events, 136 (77%) were mild and 164 (93%) were not related or unlikely related to vaccine. None of the volunteers discontinued the study due to adverse events. There were no differences in clinical laboratory parameters between study groups or trends within any study group over time (data not shown). None of the volunteers developed anti-double-stranded DNA antibodies at any timepoint throughout the study.

### Cellular Immunogenicity

IFNγ ELISpot results are summarized in Table 3. The IFNγ ELISpot responses occurred in 3/12 (25%), 4/12 (33%), and 2/12 (17%) volunteers in the low, mid and high dosage groups, respectively. There were no positive responses to any peptide pool among the placebo recipients. All but one response occurred only after the 2^nd^ or 3^rd^ vaccination.

**Table 3:** IFNγ ELISpot Results.

ADVAX dosage groups	0.2 mg	1.0 mg	4.0 mg
Positive volunteers	3/12 (25%)	4/12 (33%)	2/12 (17%)
SFC - median	240	85	68
SFC - range	88–1810	54–110	66–73
Gag responders	1	0	1
Env responders	1	3	1
Pol responders	1	0	0
Nef-Tat responders	0	1	0
Response Timing–median (week)	14	6	14
Response Timing–range (week)	1–52	6–52	14

**Table 3** summarizes the IFNγ ELISpot response rate and magnitude in spot forming cells per million PBMCs (SFC) among volunteers receiving ADVAX by dose group. There were no positive responses in placebo recipients. The timing of IFNγ ELISpot responses and distribution of antigens eliciting these responses are listed.

Polyfunctional cytokine-specific responses in the range of 0.4–0.96% were detected for only one ELISpot-positive donor, a subject in the low dosage group at week 28, 16 weeks after the last vaccination. These responses occurred in both CD3+CD4+ T cells and CD3+CD8+ T cells and showed background subtracted IFNγ, MIP-1β and TNF-α responses to one polymerase pool in the range of 0.4–0.96%, corresponding with a high ELISpot response to the same pool. The remaining intracellular cytokine assay responses from ELISpot-positive volunteers were below the limit of detection of the flow cytometry assay.

### Humoral Immunogenicity

None of the volunteers developed binding antibodies to Clade C gp120 following vaccination. Similarly, none of the volunteers tested positive on clinical HIV-1 ELISA or Western Blot at any time throughout the study.

## Discussion

This study was the first clinical trial of ADVAX in humans. Three vaccinations with ADVAX were well-tolerated at all three dosage levels, with minimal local and systemic reactogenicity.

Cellular immunogenicity, as measured by IFNγ ELISpot assays, was generally modest, sporadic, and transient, with no apparent dose response, which is in contrast to the stronger responses observed in small animals [5]. Responses occurred after the second or third vaccination in all but one volunteer who formed a transient response to Gag after the first vaccination. This relatively modest response is concurrent with other stand-alone intramuscular DNA vaccines, which have proven weakly immunogenic in humans [14]–[16].

Given that DNA vaccines provide synergistic priming of the cellular immune response when boosted by viral vaccines, the IFNγ ELISpot assay does not adequately measure the ability to prime the immune system. The magnitude of the ELISpot response also does not necessarily correlate with a protective immune response either in non-human primates [17] or in the recent STEP trial of an adenoviral-based vaccine [18], [19]. In our hands we have seen that the 16 hour detection platform of the ELISpot is more sensitive for IFNγ detection than the 6 hour detection platform of the flow cytometry assay, which may account for the paucity of detectable responses on intracellular cytokine staining. The mechanism of priming by DNA vaccines remains to be elucidated. Because the correlates of protection to HIV remain unknown, the relevance of the IFNγ ELISPOT and other assays ultimately remains unknown.

One volunteer in the low dosage group formed a particularly robust response to polymerase after the third vaccination, which was of high magnitude and sustained for at least nine months following vaccination. This was the same volunteer who formed polyfunctional antigen-specific T cell responses after vaccination. After unblinding, it was noted that this volunteer was a homosexual male who had a history of sexual relations with a long-term HIV-infected partner several years prior to enrolling in the trial. This volunteer remains HIV uninfected, and qualified for enrollment into the trial with a negative HIV ELISA, no bands on HIV western blot, and undetectable viral load. One explanation is that the robust response to polymerase after vaccination with ADVAX may reflect a “boosting” effect by ADVAX after “priming” with exposure to HIV in the past, as described previously [20]. It is also possible that this response is a non-specific cross reaction to both polymerase peptide pools. However, this finding may have implications for assessment of responses to HIV vaccines in high-risk, uninfected populations who may have prior immunologic exposure to HIV not detected by conventional screening assays. Further immunologic characterization of this volunteer pre- and post-vaccination is ongoing.

The fact that no antibody responses were detected is disappointing, but consistent with the performance of other stand-alone DNA vaccines delivered to date [15]. Other Clade C based HIV-1 vaccine candidates have been tested in DNA prime, Viral vector boost combinations [9], [21], [22]. To test the priming ability of ADVAX, two clinical trials are now being conducted in the United Kingdom and in India, respectively, where 2 or 3 doses of 4 mg of ADVAX, either administered by Biojector ® 2000 or regular intramuscular needle injection, are given as prime followed by a recombinant multigenic MVA expressing HIV-1 Clade C env, gag, RT, rev, tat and nef genes [23]. Attempts to improve the immunogenicity of DNA vaccines alone are also underway through improvements in DNA vaccine delivery or use of adjuvants [13], [24]. ADVAX is currently in a Phase I clinical trial to assess safety and immunogenicity when delivered by *in vivo* electroporation with the Tri Grid™ Delivery System [25].

## Supporting Information

Checklist S1 (0.19 MB DOC)

Protocol S1 (3.74 MB PDF)
