# Supplementary material for: Phase 1 Safety and Immunogenicity Evaluation of ADVAX, a Multigenic, DNA-Based Clade C/B' HIV-1 Candidate Vaccine
Source: PLoS One. 2010 Jan 25;5(1):e8617. doi: 10.1371/journal.pone.0008617 (PMC2799527; doi:10.1371/journal.pone.0008617)
Supplement: Protocol S1 — (3.74 MB PDF) [file pone.0008617.s002.pdf]

DNA (ADVAX) Dose-Escalating Study

Protocol Title: A Randomized, Placebo-Controlled, Dose-Escalating, Double-Blinded Phase 1 Study to Evaluate the Safety and Immunogenicity of a Clade C DNA Vaccine ADVAX e/g + ADVAX p/n-t (ADVAX) Administered Intramuscularly to HIV-Uninfected, Healthy Volunteers.

Protocol Number: IAVI C001

Phase: Phase 1

Sponsor: International AIDS Vaccine Initiative (IAVI)
110 William Street, 27th Floor
New York, New York
10038-3901
USA

Date of Protocol Version 1: 8 September 2003

Date of Amendment Version 1.1: 22 December 2003

UNCONTROLLED COPY

SYNOPSIS

**TITLE:**	A Randomized, Placebo-Controlled, Dose-Escalating, Double-Blinded Phase 1 Study to Evaluate the Safety and Immunogenicity of a Clade C DNA Vaccine ADVAX e/g + ADVAX p/n-t (ADVAX) Administered Intramuscularly to HIV-Uninfected, Healthy Volunteers.
**INVESTIGATOR SITES:**	Aaron Diamond AIDS Research Center (ADARC)<br>The Rockefeller University Hospital<br>The Rockefeller University, 1230 York Avenue<br>New York, New York 10021<br><br>University of Rochester Medical Center<br>601 Elmwood Ave.<br>Rochester, NY 14642
**PHASE:**	Phase 1
**SPONSOR:**	International AIDS Vaccine Initiative (IAVI)<br>110 William Street, 27th Floor<br>New York, New York<br>10038-3901<br>USA
**OBJECTIVES:**	***Primary:***<br><br>To evaluate the safety of ADVAX e/g + ADVAX p/n-t administered three times, intramuscularly<br><br>To evaluate the safety of three dosage levels of ADVAX e/g + ADVAX p/n-t<br><br>***Secondary:***<br><br>To evaluate the immunogenicity of ADVAX e/g + ADVAX p/n-t administered three times, intramuscularly<br><br>To evaluate the immunogenicity of three dosage levels of ADVAX e/g + ADVAX p/n-t<br><br>To explore the breadth of the immune responses to different virus strains

SYNOPSIS

STUDY DESIGN TABLE:

Group	Vaccine/ Placebo	Week 0 *	Week 4*	Week 12*
Low	12/3	0.2 mg	0.2 mg	0.2 mg
Middle	12/3	1.0 mg	1.0 mg	1.0 mg
High	12/3	4.0 mg	4.0 mg	4.0 mg
Total Number	36/9			

* ADVAX e/g +ADVAX p/n-t or Placebo, given IM at the dosage indicated.

STUDY POPULATION:

Healthy male or female adults 18-60 years of age (with no more than 10% of volunteers at each site over the age of 45), willing to undergo HIV testing, use an effective method of contraception and who, in the opinion of the principal investigator or designee, understand the study and can provide written informed consent.

Principal exclusion criteria include infection with HIV-1 or HIV-2, reported high risk behaviour for HIV infection, pregnancy and lactation, chronic disease, recent vaccination or receipt of a blood product or experimental agent, and previous severe local or systemic reaction to vaccination or history of severe allergic reactions.

NUMBER OF VOLUNTEERS:

Approximately 45 volunteers (36 vaccine recipients/9 placebo recipients) will be enrolled in the study. An over-enrollment of up to 10% (5 additional volunteers) will be permitted in the study.

DESCRIPTION OF INVESTIGATIONAL PRODUCT:

ADVAX e/g + ADVAX p/n-t contains Clade C HIV env and gag (eg) and Clade C HIV pol and nef/tat fusion (pnt) mixed in a 1:1 ratio.

The Placebo is 0.01M sodium phosphate, 150mM NaCl, pH 7.2.

Vaccine/ Placebo	Dosage Level	Total Volume in Vial (ml)	Total Injected Volume (ml)	Route of Administration
ADVAX e/g + ADVAX p/n-t	0.2mg	1.2ml	1.0ml	IM
	1.0mg	1.2ml	1.0ml	IM
	4.0mg	1.2ml	1.0ml	IM
Placebo	NA	1.2ml	1.0ml	IM

IM= Intramuscular

SYNOPSIS

**DOSE ESCALATION:**	15 volunteers will be enrolled in each of up to three dosage groups and randomized in a 4:1 ratio of active vaccine to placebo. Safety and tolerability of the ADVAX e/g + ADVAX p/n-t investigational product will be evaluated at least 14 days after the tenth volunteer in the lower dosage group receives the second injection before proceeding to the middle dosage group. Likewise, there will be an evaluation at least 14 days after the tenth volunteer in the middle dosage group receives the second injection before proceeding to the higher dosage group.
**BLINDING:**	Site personnel and volunteers will be blinded only with respect to the allocation of placebo or active vaccine. Blinding will not apply to the assignment of dosage groups (lower, middle or higher).
**DURATION OF STUDY PARTICIPATION**	Volunteers will be screened up to 42 days before vaccination and will be followed for 18 months after the first vaccination.
**PRIMARY ENDPOINTS:**	***Safety and Tolerability:***<br>• Severe and very severe local reactogenicity events (pain, tenderness, erythema/skin discoloration, edema, skin damage [vesiculation/ulceration], induration, formation of crust or scab)<br>• Severe and very severe systemic reactogenicity events (fever, chills, headache, nausea, vomiting, malaise, myalgia, arthralgia, rash)<br>• Severe and very severe other adverse events (including laboratory abnormalities)<br>• All Serious Adverse Events<br>• Proportion of volunteers with mild and moderate local and systemic reactogenicity events and adverse events
**SECONDARY ENDPOINTS:**	***Safety and Tolerability:***<br>• Four-fold increase in antibody titer to double-stranded DNA with the titer reaching a level defined by the local laboratory as abnormal<br>***Immunogenicity:***<br>• HIV-1 specific T- cell responses quantified by ELISPOT<br>• HIV-1 specific T-cell responses quantified by cytokine flow cytometry (CFC)<br>• Binding and neutralizing antibody responses

SYNOPSIS	
	• All immune responses will be evaluated for proportion of responders and the mean responses will be compared
**EVALUATION FOR INTERCURRENT HIV INFECTION:**	Volunteers will be tested for HIV-1 and HIV-2 antibodies by ELISA according to the Schedule of Procedures (Appendix A). If the ELISA test is positive, a pre-defined testing algorithm will be followed to determine whether antibodies have been induced by the vaccine or whether the volunteer has become infected with HIV through exposure in the community. The result will be communicated as HIV infected/HIV non-infected.<br>HIV testing at additional time points may be performed at the discretion of the volunteer and Investigator as medical or social circumstances arise.
**STATISTICAL CONSIDERATIONS:**	Data will be entered into the clinical trial database and will be available to designated site personnel in a blinded form.<br>At the end of the study, a full analysis will be prepared according to a pre-specified statistical analysis plan.<br>Safety and tolerability will be addressed by examining the overall rates of severe and very severe reactogenicity events and other adverse events and SAEs that might be associated with vaccination and the number of volunteers who experience these events. All clinical and routine laboratory data will be included in the safety analysis.<br>Immunogenicity analyses will primarily be restricted to volunteers who complete the vaccination schedule. Volunteers will be classified as responders or non-responders based on the results of the immunological assessments.

UNCONTROLLED COPY

Table of Contents

1.0 CONTACT INFORMATION 10

1.1 Site Contact Information (Principal Investigators) 10

1.2 Sponsor Contact Information 10

2. 0 SIGNATURE PAGE 12

3.0 INTRODUCTION AND BACKGROUND 13

3.1 DNA Vaccines 13
3.1.1 The candidate DNA.HIV vaccine ADVAX (ADVAX e/g + ADVAX p/n-t) 13
3.1.2 Pre-clinical studies with DNA vaccines 15
3.1.3 Preclinical Studies with ADVAX e/g+p/n-t 17
3.1.4 Human trials with DNA vaccines 18

4.0 STUDY OBJECTIVES 19

4.1 Primary Objectives 19

4.2 Secondary Objectives 19

5.0 STUDY DESIGN AND ENDPOINTS 20

5.1 STUDY ENDPOINTS 20
5.1.1 Primary Endpoints 20
5.1.2 Secondary Endpoints 20

5.2 STUDY DESIGN 20
5.2.1 Duration of the Study 21
5.2.2 Study Population 21
5.2.3 Inclusion Criteria 21
5.2.4 Exclusion Criteria 22
5.2.5 Recruitment of Study Volunteers 23

6.0 STUDY VISITS 23

6.1 Screening Visit 23

6.2 Vaccination Visit Procedures 24

6.3 Post-Vaccination Follow-up Visits 24

6.4 Additional Follow-up Visits 24

6.5 Final Visit/Early Termination Visit 25

7.0 STUDY VACCINE AND PLACEBO 25

7.1 Description of Investigational Product 25

7.2 Storage and Shipment of Investigational Product 25

7.3 Dispensing and Handling of Investigational Product 25

7.4 Administration of Investigational Product 26

7.5 Accountability and Disposal of Used and Unused Investigational Product 26

8.0 STUDY PROCEDURES 26

8.1 Informed Consent 26

8.2 Medical History and Physical Examination 27

8.3 HIV Testing and Counseling 27

8.4 Family Planning Counseling 27

8.5 Blood Collection and Shipment 27

8.6 Reimbursement 28

8.7 Randomization and Blinding 28

8.8 Unblinding Procedure 28

9.0 ASSESSMENTS 28

9.1 Safety Assessments 28
9.1.1 Local reactogenicity events 28
9.1.2 Systemic reactogenicity events 29
9.1.3 Other adverse events 29
9.1.4 Routine laboratory parameters 29

9.2 Immunogenicity Assessments 30
9.2.1 Antibody responses 30
9.2.2 Cellular responses 30

9.3 Other Assessments 30
9.3.1 HLA typing 30
9.3.2 HIV antibody test 30
9.3.4 Pregnancy test 30
9.3.5 Concomitant medication 30

10.0 ADVERSE EVENTS 30

10.1 Definition 30

10.2 Severity Grading of Adverse Events 31

10.3 Serious Adverse Events (SAEs) 31

10.4 Relationship to Study Product 32

10.5 Collection of adverse events 33

10.6 Reporting (Serious) Adverse Events 33

10.7 Clinical Management 34

UNCONTROLLED COPY

10.8 Pregnancy 35

10.9 Intercurrent HIV Infection 35

11.0 MANAGEMENT OF HIV ISSUES DURING AND FOLLOWING THE TRIAL 35

11.1 HIV Testing 35

11.2 HIV Infection 35
11.2.1 Counseling 35
11.2.2 Referral for support and/or care 36

11.3 Social Discrimination as a Result of an Antibody Response to Vaccine 36

12.0 DISCONTINUATION OF VACCINATION AND/OR VOLUNTEER WITHDRAWAL FROM STUDY 36

12.1 Discontinuation of Further Vaccinations 36
12.1.1 Follow up after discontinuation of further vaccinations 37

12.2 Withdrawal from the Study (Early Termination) 37
12.2.1 Follow up after withdrawal from study (Early Termination) 37

13.0 DATA HANDLING 38

13.1 Data Handling at the Trial Sites 38

13.2 Data Collection and Transfer at the IAVI Core Laboratory 38

13.3 Data Entry at the Trial Sites 38

13.4 Quality Assurance and Audit 38

13.5 Data Handling and Analysis 38

13.6 Trial Supervision and Monitoring 39

14.0 STATISTICAL CONSIDERATIONS 39

14.1 Sample Size 39

14.2 Statistical Power and Analysis 39

15.0 DATA AND BIOLOGICAL MATERIAL 41

16.0 ADMINISTRATIVE STRUCTURE 41

16.1 Trial Steering Committee (TSC) 41

16.2 Data and Safety Monitoring Board (DSMB) 41
16.2.1 Content of interim review 41
16.2.2 Indications for discontinuation of vaccinations in all volunteers 42

17.0 INDEMNITY 42

18.0 PUBLICATION 42

UNCONTROLLED COPY

19.0 ETHICAL CONSIDERATIONS 42

GLOSSARY 43

REFERENCES 44

APPENDIX A: SCHEDULE OF PROCEDURES 47

APPENDIX B: SAMPLE INFORMED CONSENT FORM 48

APPENDIX C: ADVERSE EVENT GRADING TOXICITY TABLE 55

APPENDIX D: LABORATORY NORMAL VALUES (SITE SPECIFIC) 63

UNCONTROLLED COPY

1.0 CONTACT INFORMATION

1.1 Site Contact Information (Principal Investigators)

David D. Ho, MD
Director and CEO
Aaron Diamond AIDS Research Center
455 First Avenue, 7th Floor
New York, N Y 10016

Michael C Keefer, MD
Box # 689 (Room # 3-6206)
University of Rochester Medical Center
601 Elmwood Ave.
Rochester, NY 14642

1.2 Sponsor Contact Information

<u>Trial Sponsor</u>

International AIDS Vaccine Initiative (IAVI)
110 William Street, 27th Floor
New York, NY 10038-3901 USA

<u>Medical Monitor</u>

Soe Than, MD, PhD
International AIDS Vaccine Initiative (IAVI)
110 William Street, 27th Floor
New York, NY 10038-3901, USA

<u>Sponsor's Director of Medical Affairs</u>

Patricia Fast MD, PhD
Director, Medical Affairs
International AIDS Vaccine Initiative (IAVI)
110 William Street, 27th Floor
New York, NY 10038-3901, USA

<u>Data Coordinating Center</u>

The EMMES Corporation
401 N. Washington Street
Suite 700
Rockville, Maryland
20850, USA

UNCONTROLLED COPY

Trial Monitoring

Phumla Adesanya
Clinical Research Associate
International AIDS Vaccine Initiative (IAVI)
110 William Street, 27th Floor
New York, NY 10038-3901, USA

Core Laboratory

Professor Frances Gotch
Department of Immunology, Faculty of Medicine, Imperial College
Chelsea and Westminster Hospital
369 Fulham Road
London SW10 9NH

Jill Gilmour, Ph.D
Director, Clinical Immunology, Core Laboratory
International AIDS Vaccine Initiative (IAVI)
110 William Street, 27th Floor
New York, NY 10038-3901, USA

Tony Tarragona, Ph.D.
Lab Manager/Administrator
IAVI Core Lab
Imperial College London, Faculty of Medicine
St. Stephen's Centre, 5th Floor
Chelsea Campus
369 Fulham Road
London SW10 9NH

2. 0 SIGNATURE PAGE

The signatures below constitute the approval of this protocol and the appendices, and provide the necessary assurances that this trial will be conducted in compliance with the protocol, GCP and the applicable regulatory requirement(s).

Sponsor:

Signed: ______________________________ Date: ______________

Patricia Fast, MD, PhD
Director, Medical Affairs, IAVI

Principal Investigator:

Signed: ______________________________ Date: ______________

Name (please print) :______________________

Name of Institution (please print):

____

3.0 INTRODUCTION AND BACKGROUND

In June 1981, the Centre for Disease Control (CDC) in the United States reported the first clinical evidence of a disease that would become known as Acquired Immunodeficiency Syndrome (AIDS). Twenty years later, the AIDS epidemic has spread all over the world. Since the beginning of the epidemic, 65 million people have been infected. Globally, over 40 million people are today living with HIV infection, with over 5 million new infections acquired annually in 2001[1]. More than 25 million individuals have lost their lives to the disease; in 2001, 3 million people died of AIDS. More than 90% of new HIV infections occur in developing countries, with the majority of infections found in Sub-Saharan Africa and South East Asia[1]. There is an urgent need to explore approaches to control the epidemic, in particular, preventive measures such as health education, treatment of sexually transmitted diseases, preventive vaccines and topical microbicides.

The HIV vaccine strategies that are currently being investigated in clinical trials include: proteins and peptides ("subunits"), live vector-based vaccines and DNA vaccines. Subunit vaccines, i.e., highly purified recombinant HIV-1 envelope proteins or synthetic peptides, have to date not elicited strong virus-specific CTL nor antibody responses that can neutralize primary isolates of HIV-1, even when adjuvanted with potent immunostimulants[2-4]. New vectors, such as an attenuated live virus or bacteria carrying genetic material representing a portion of the HIV[3-5], may prove useful for HIV vaccines. Another new approach has been the use of genetically engineered plasmid DNA to direct the synthesis of an immunogen within the host cells[6-9].

Theoretically, an ideal HIV vaccine would elicit HIV-specific CD8+ cytotoxic T-lymphocytes (CTL) and also antibodies capable of neutralizing primary HIV isolates. This protocol proposes studies of a vaccine approach designed to induce cell-mediated immune (CMI) responses, which are thought to slow the replication of HIV and destroy HIV-infected cells.

3.1 DNA Vaccines

DNA vaccines contain a gene encoding one or more antigens under the regulation of a eukaryotic enhancer/promoter and polyadenylation signals that confer appropriate expression of the antigens. DNA encoding a selected component of a pathogen is injected as a plasmid. The identity of the immunogen gene is determined by sequencing. When injected into muscle, cells surrounding the injection site internalize the plasmid and transport the DNA to the nucleus where transcription, translation and post-translational modification occur as they would in natural infection. The feasibility of genetic vaccination has been shown in several experimental model systems [12-14]. Nucleic acid vaccines may elicit both antibody and CTL responses[14-18].

3.1.1 The candidate DNA.HIV vaccine ADVAX (ADVAX e/g + ADVAX p/n-t)

ADVAX is a DNA vaccine consisting of two vectors (ADVAX e/g and ADVAX p/n-t) based on pVAX1, a commercially-available plasmid from Invitrogen®. pVAX1 was designed specifically for use in the development of DNA vaccines, and was constructed to be consistent with United States Food and Drug Administration (FDA) guidelines[19]. It was modified by inserting an additional promoter human elongation factor 1α (hEF1α). This alteration of pVAX1, yielding pADVAX (Figure 3.1.1.1), permits independent, high-level expression of a second genetic insert.

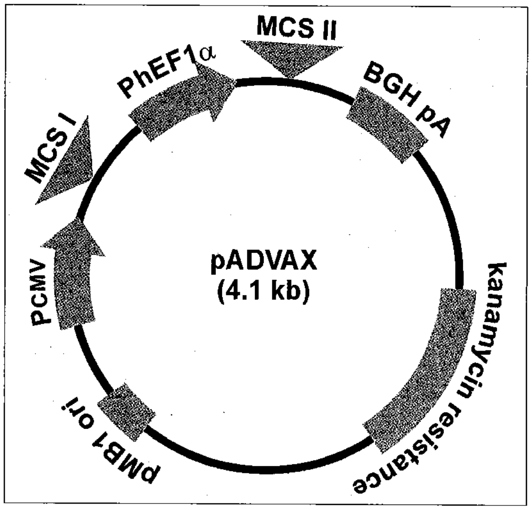

Figure 3.1.1.1: Schematic map of pADVAX

Of note, the bicistronic capacity of pADVAX is more potent (by 10- to 20-fold) than that achieved with use of an internal ribosomal entry site, or IRES[20]. One of the vectors (ADVAX e/g) contains HIV-1 *env* and *gag* that have been modified for optimal for mammalian expression. Codon optimization represents a facilitation of Rev/RRE-independent nuclear export[21,22], and is consistently found to enhance expression of viral genes. Overlapping PCR was used to unite oligonucleotides (80- to 90-mers overlapping by 16-18) with sequences reflecting this ideal codon selection. Gene expression was enhanced by 100- to 1000-fold as measured by ELISA or western blot. Genes were further modified by incorporating a tissue plasminogen activator (tPA) leader sequence (amino acids: MDAMKRGLCCVLLLCGAVFVSAR), replacing the native sequence of *env* and supplementing the *gag* gene. Of note, this sequence is thought to enhance expression in part by facilitating transport of protein from the endoplasmic reticulum (ER) to the Golgi apparatus [23-26]. With this refinement, gene expression was further enhanced by 3- to 5-fold.

The second vector (ADVAX p/n-t) was constructed along similar lines. Overlapping PCR was used to unite "codon-optimized" oligonucleotides for synthesis of *pol*, *nef* and *tat*. This time, however, additional measures were taken to ensure safety for *in vivo* use. To prevent polypeptide processing a deletion was made in the active site of protease (PR) in the *pol* gene. Additionally, a point mutation was also made in the active site of reverse transcriptase (RT). To be able to incorporate all three genes into a single pADVAX-based vector, a *nef-tat* fusion gene, was constructed by overlapping PCR. All genetic sequences were kept intact, thereby preserving all immunogenic epitopes in the resultant fusion protein. As before, we added a tPA leader sequence to both *pol* and *nef-tat*.

The ADVAX vaccine, then, involves two dual-promoter vectors: ADVAX e/g, which expresses *env* and *gag*, and ADVAXp/n-t , which expresses *pol* and *nef-tat* (Figure 3.1.1.2). All five HIV-1 genes carried by these two vectors have been evaluated thoroughly *in vitro* to ensure expressive potential and safety in the context of these unique plasmid vectors.

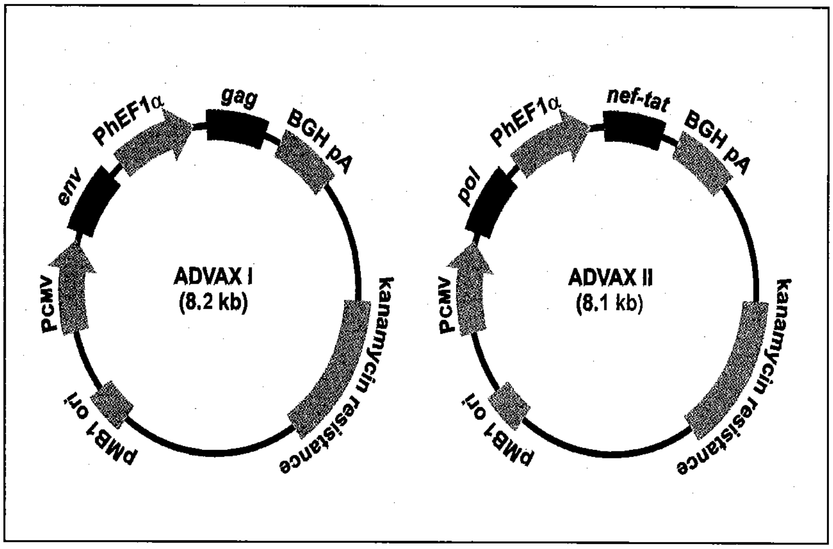

Figure 3.1.1.2: Schematic map of ADVAX e/g (ADVAX I) and ADVAX p/n-t (ADVAX II)

3.1.2 Pre-clinical studies with DNA vaccines

In animal models, DNA vaccination has been shown to induce protective immunity against a variety of viral, bacterial, and parasitic pathogens. Plasmid DNA expressing genes derived from HIV was recently shown to induce humoral and cellular immune responses in rodents[12,13,27] and non-human primates[14,15]. No local reactions have been seen in animals.

There is a theoretical concern regarding the possible integration of DNA vaccine plasmid into the genome of a small number of somatic cells of the person being vaccinated. The concern is that an integrated vaccine may result in insertional mutagenesis through activation of oncogenes or inactivation of tumor suppressor genes. In addition, an integrated plasmid DNA vaccine may result in chromosomal instability through the induction of chromosomal breaks or rearrangements. It is unclear what frequency of integration is related to development of disease. Not every single process of integration will lead to insertional mutagenesis, meaning activation of an oncogene or functional deletion of a tumor suppressor gene[36]. The induction of malignant tumors requires a series of genetic events, which have to occur in addition to insertional mutagenesis.

The framework for regulating DNA vaccines has been in place since the first clinical trial was initiated in the mid-1990s. American and European regulatory guidance has evolved on the basis of insights provided by ongoing pre-clinical and clinical studies. These studies include analyses of the safety of DNA vaccines in normal volunteers, and recent data concerning the tissue distribution, persistence, and integration of DNA plasmids. Kurth[37] hypothesized that there was a risk of possible negative event in 10^6 healthy volunteers as a result of DNA vaccination. Temin et al[28] estimated that the probability of injected DNA having a harmful effect is less than 10^{-16} to 10^{-19} / DNA molecule.

Pre-clinical studies in mice, rabbits, guinea pigs have been performed with different plasmid DNA vaccines such as malaria, HIV and HPV vaccines. Routes of administration included intramuscular, intravenous, per-cutaneous (Biojector). Aluminum phosphate as adjuvant was tested under experimental conditions.

In pre-clinical safety studies, repeated intramuscular injections had no adverse effects on clinical chemistry, or hematology, and did not result in any organ pathology or systemic toxicity. No evidence of autoimmune-mediated pathology, anti-nuclear antibodies (ANA) or

antibodies to dsDNA were observed [39]. Distribution and integration studies demonstrated that six weeks following intramuscular injection, the vast majority of the plasmid was detected near the injection site. Lower levels of plasmid were also detected in draining lymph nodes. At early time points (1-7 days) after injection, a low level of systemic exposure could be detected. Occasionally, plasmid was detected in gonads, but it dissipated rapidly and was extrachromosomal[40].

A quantitative integration assay has shown that all detectable plasmid in treated mouse quadriceps was extrachromosal[41].

It seems unclear whether sequences of plasmid DNA are covalently linked to genomic DNA or simply adventitiously associated with genomic DNA. If an assumption is made that the highest level plasmid DNA found associated with genomic DNA represented covalently integrated plasmid inserts, and that each insert resulted in a mutational event, the calculated rate of mutation would be 3000 times less than the spontaneous mutation rate of 1x10^5 per gene for mammalian genomes[42].

Injection of a plasmid DNA vaccine containing the human papilloma virus HPV type 16 E7, which is known to increase integration *in vivo*, did not result in detectable integration in mice[40].

After injection of DNA into an animal, only a small proportion of the DNA molecules enter cells[43]. Most detectable plasmid remains extrachromosal[40]. The probability of any DNA molecule integrating into the chromosome is also low, and given that oncogenesis is a multifactorial event, the risk of insertional mutagenesis is exceedingly low[43].

If integration occurred, the frequency would be at least three orders of magnitude below spontaneous mutation rate. Plasmid DNA, occasionally found extrachromosomally in testes and ovaries, dissipated quickly, which indicates a low risk of germline transmission[41].

Available evidence suggests that DNA vaccines currently being tested clinically rarely integrate[44] and that the risk of mutation due to integration of plasmid DNA vaccines following intramuscular injection is negligible[41].

Other animal studies have addressed the concern that plasmid DNA would have adverse effects on organs, including gonads, or on haematological and biochemical parameters. No significant changes or toxicity were observed in murine or primate[29-31] models. Regarding autoimmunity, in several animal studies, there was no histopathological evidence of autoimmune disease nor anti-DNA antibodies[29,30]. In one study, plasmid DNA persisted in mouse skeletal muscle for at least 19 months after intramuscular injection[31].

A number of studies conducted to characterize the tissue distribution of a variety of plasmids have shown that though a low level of systemic exposure was detected at early time points, i.e. 1-7 days post intramuscular injection, the plasmid DNA at distal sites dissipated to undetectable levels by 4-6 weeks post dose [39, 40].

Manam et al. [41] published their findings characterizing the tissue distribution of plasmid DNA and impact of the variables of DNA sequence, adjuvants and delivery method on potential for integration into the host genome, using mice. The transgenes studied included an HIV-1 *gag* gene (with and without a tPA secretory signal) and the human papilloma virus type 16 E7 gene, known to increase integration *in vitro*. The results from highly sensitive PCR analysis of tissues suggest that the risk of plasmid integration following intramuscular injection is exceedingly small under a variety of experimental conditions.

Corresponding evaluation of potential integration events using sensitive kinetic PCR analysis, after isolation of high molecular weight genomic DNA from free plasmid, have shown that the persisting DNA can be separated from the genomic DNA and is not integrated. These studies have provided estimates of the integration potential of several different plasmids, and have concluded, with calculations based on worst-case error, that the number of plasmid copies quantified in the genomic DNA fraction, represents a frequency of integration lower than the rate of spontaneous mutation. This was also the conclusion of Martin et al. [42] in an earlier preclinical study of a plasmid DNA malaria vaccine.

3.1.3 Preclinical Studies with ADVAX e/g+p/n-t

Studies to assess the immunogenicity of ADVAX e/g+ADVAX p/n-t have been performed in BALB/c mice. For the purpose of gauging cell-mediated immune (CMI) responses, an ELISPOT assay was used to detect antigen-specific gamma interferon secretion by mouse splenoctyes. When three doses of plasmid DNA vaccine were administered in 3-week intervals, dose escalation experiments reveal a dose-response effect. Both CD4+ and CD8+ T cell mediated responses were induced against epitopes of all five plasmid-encoded HIV-1 immunogens, i.e. Env, Gag, Pol, Nef and Tat.

Humoral immune responses were also observed when anti-Gag antibody titers were measured in mice immunized with ADVAX e/g alone. Serum samples from immunized mice also demonstrate an antibody response to Env by western blot analysis.

In an evaluation of several prime-boost regimens using ADVAX plasmid DNA and ADMVA candidate vaccines, the DNA prime + MVA boost regimen induced the strongest CMI responses to peptides representing epitopes expressed by the five HIV-1 transgenes.

Studies were conducted to assess the potential systemic toxicity and reactogenicity of ADVAX e/g and ADVAX p/n-t in New Zealand White rabbits using the intramuscular route of administration, the intended route for clinical studies. All pre-clinical studies have been conducted in compliance with current U.S. FDA Good Laboratory Practice (GLP) Regulations for Non-clinical Laboratory Studies (21 CFR Part 58) to support safety claims. A 98-day repeat dose toxicity study was conducted at dose levels of 4 mg, 1 mg or vehicle (PBS), dosing at 4-week intervals. Twenty rabbits, ten females and ten males, were treated at each dose level. A total of four doses were administered over a 3 month period, representing one additional dose beyond the proposed clinical dosing regimen of 0, 1, and 3 months. The 4 mg dose level represents the high clinical dose proposed in the initial Phase I trial. Routine safety parameters were measured, including mortality, body weight gain, food consumption, clinical observations, ophthalmoscopy, local reactogenicity, clinical chemistry, hematology, and gross and microscopic histopathology at necropsy. Microscopic histopathological assessment was limited to the high dose group, i.e. 4 mg and control group. Interim necropsy of half of the animals in each group occurred at 48 hours post final dose, while the balance of animals were considered a recovery group, with final necropsy at 2 weeks post final dose.

Based upon the results of this study, intramuscular administration of up to 4 x 4 mg doses of ADVAX e/g+ADVAX p/n-t plasmid DNA vaccine over a 98-day interval was well tolerated in laboratory animals with no test article-related findings in mortality, clinical signs of toxicity, body weights, body weight changes, food consumption, ophthalmology, clinical pathology or gross pathology.

All but 2 of 52 animals survived until their scheduled termination and there were no adverse clinical observations noted that were attributed to treatment with the plasmid DNA vaccine. One male rabbit (1 mg) was euthanized due to luxation in the lumbar region, the result of handling the animal after scheduled blood sampling. One early death (female, 4 mg dose group) occurred 7 days after the initial dose. Prior to death on SD 8, the animal exhibited diarrhea, vaginal discharge, loss of appetite and lost 606 grams of body weight in a 7 day interval. Loss of appetite in this animal was evident at the initiation of dosing. Dehydration caused by the pre-existing condition of mucoid enteritis, an enteropathy of unknown etiology affecting rabbits, was considered to be the probable cause of death in this animal, given that anorexia and mucoid diarrhea were immediately evident at the initiation of the study.

Microscopic histopathological evaluation of 37 tissues from every high dose and control animal indicated that all were within histologic limits of normalcy for laboratory rabbits of the age and strain used in the study.

A second GLP study was conducted to determine the tissue distribution of a single intramuscular injection of the plasmid DNA vaccine and evaluate the persistence of the plasmids in a selected panel of tissues over a period of 60 days post-injection. Body weight gains, clinical observations and food consumption were also evaluated in this study. A single intramuscular injection of 200µg of ADVAX e/g+ADVAX p/n-t was administered to thirty CD-1 mice, an outbred strain. Mice in the control group received an equivalent volume of vehicle (PBS). At 3, 30 and 60 days post-injection, a subset of treated and control mice were sacrificed and specified tissues were removed for DNA extraction. Specifically blood, gonads, brain, lung, heart, spleen, kidney, liver, bone marrow, lymph nodes and the quadriceps muscle at the injection site were harvested at necropsy. Extracted DNA from tissues were analyzed for the presence of plasmid DNA sequences using a sensitive, plasmid-specific, qualified PCR assay. The quantitative PCR assay utilized primer and probe sets specific for the human elongation factor 1 alpha promoter and human CMV promoter sequences found in ADVAX e/g and ADVAX p/n-t plasmids. The design of the GLP biodistribution study provides the data necessary to evaluate intramuscular administration of ADVAX plasmids for potential aberrant tissue distribution and to define the kinetic profile of persistence of the plasmids, particularly as it relates to the potential for dissemination of plasmid DNA to the germ line.

No treatment related changes in mortality, clinical signs of toxicity, body weights, body weight changes, or food consumption were observed in CD-1 mice treated with ADVAX e/g+ADVAX p/n-t administered intramuscularly in the 60-day study. Analysis of integration potential using quantitative Polymerase Chain Reaction (qPCR) assays targeting the human cytomegalovirus (CMV) promoter and human elongation factor promoter DNA sequences, in combination with field inversion gel electrophoresis purification, indicated no evidence of detectable plasmid DNA integration into host genomic DNA.

3.1.4 Human trials with DNA vaccines

Several studies have been performed in humans with DNA vaccines other than HIV.DNA vaccines. Anti-malaria DNA vaccines administered intramuscularly induced some CTL and no antibody responses, and were well tolerated up to 2.5 mg per dose [32,33]. A pilot study (Protocol 400-003-03) with an HIV env/rev DNA construct was conducted in HIV-uninfected volunteers at the National Institutes of Health (NIH) Clinical Centre, USA. Doses as high as 3 mg were given as a single injection. Adverse events were mild and infrequent and T-cell responses were observed[34].

A Phase 1 clinical study (AVEG Protocol 031) with an HIV-1 gag/pol DNA vaccine was performed in 52 HIV-1 uninfected adult volunteers aged 18-60 years with low risk for HIV-1 infection. Doses as high as 3 mg at 0, 1, 3 and 6 months were given. This vaccine was able to induce antigen-specific proliferation and production of chemokines by PBMC of vaccinated individuals, *in vitro*. Preliminary data on this HIV-1 gag-pol DNA vaccine were presented by Tellez et al[35].

In early 2000, Merck began a human trial of a DNA vaccine using HIV gag with the intention to boost with recombinant adenovirus. Preliminary data have been presented documenting safety and immunogenicity (Emini, CROI IX, Seattle, Washington, 2002).

A candidate DNA.HIVA vaccine has undergone Phase 1 clinical trials through the collaboration of IAVI, the Medical Research Council in Oxford, UK and with the Kenya Aids Vaccine Initiative (KAVI) in Nairobi, Kenya. The DNA.HIVA vaccine was given to a total of 31 volunteers at either 100μg (n=6) or 500 μg (n=25) intramuscularly. It has been well tolerated and immunogenic. Local side effects observed in the UK trial were mild to moderate, occurred mostly on the first post-vaccination day and resolved by 48 hours. Systemic side effects were mild to moderate and resolved within 1-3 days. In the Kenya trial no local or systemic side effects have been observed. A Phase 1 study testing a prime-boost approach and enrolling 120 volunteers started at the beginning of April 2002 in the UK. Dosage levels up to 2 mg DNA.HIVA per injection are being tested. As of May 19, 2003 approximately 103 individuals have been enrolled. No pattern of risk has been found among reported events. Thus far the vaccine has been found to be generally safe and well tolerated.

4.0 STUDY OBJECTIVES

4.1 Primary Objectives

- To evaluate the safety and tolerability of ADVAX e/g + ADVAX p/n-t administered three times, intramuscularly
- To evaluate the safety and tolerability of three dosage levels of ADVAX e/g+ADVAX p/n-t

4.2 Secondary Objectives

- To evaluate the immunogenicity of ADVAX e/g + ADVAX p/n-t administered three times, intramuscularly
- To evaluate the immunogenicity of three dosage levels of ADVAX e/g + ADVAX p/n-t
- To explore the breadth of the immune responses to different virus strains

5.0 STUDY DESIGN AND ENDPOINTS

5.1 STUDY ENDPOINTS

5.1.1 Primary Endpoints

Safety and Tolerability:

- Severe and very severe local reactogenicity events (pain, tenderness, erythema/skin discoloration, skin damage [vesiculation/ulceration], induration, formation of crust or scab)
- Severe and very severe systemic reactogenicity events (fever, chills, headache, nausea, vomiting, malaise, myalgia)
- Severe and very severe other adverse events (including laboratory abnormalities)
- All Serious Adverse Events
- Proportion of volunteers with mild and moderate local and systemic reactogenicity events and adverse events

5.1.2 Secondary Endpoints

Safety and Tolerability:

- Four-fold increase in antibody titer to double-stranded DNA with the titer reaching a level defined by the local laboratory as abnormal

Immunogenicity:

- HIV-1 specific T-cell responses quantified by ELISPOT
- HIV-1 specific T-cell responses quantified by cytokine flow cytometry (CFC)
- Binding and neutralizing antibody responses
- All immune responses will be evaluated for proportion of responders and the mean responses will be compared

5.2 STUDY DESIGN

The study is a randomized, dose-escalating, double blind (with respect to active vaccine or placebo), placebo-controlled study.

Safety and tolerability of the ADVAX e/g + ADVAX p/n-t vaccine/placebo will be evaluated at least 14 days after the tenth volunteer in the lower dosage group receives the second injection before proceeding to the middle dosage group. Likewise, there will be an evaluation at least 14 days after the tenth volunteer in the middle dosage group receives the second injection before proceeding to the higher dosage group.

Table 5.2
Study Design

Group	Vaccine/Placebo	Week 0*	Week 4*	Week 12*
Low	12/3	0.2 mg	0.2 mg	0.2 mg
Middle	12/3	1.0 mg	1.0 mg	1.0 mg
High	12/3	4.0 mg	4.0 mg	4.0 mg
Total Number	36/9	* ADVAX e/g +ADVAX p/n-t or Placebo given IM at the dose indicated		

5.2.1 Duration of the Study

Volunteers will be screened up to 42 days before vaccination and will be followed for 18 months after the first vaccination.

5.2.2 Study Population

The study population comprises healthy volunteers who are not infected with HIV-1 or HIV-2, are 18 to 60 years of age (not more than 10% of the volunteers at each site may be over 45 years of age), and have not reported high-risk behaviour for HIV infection.

Approximately 45 volunteers (36 vaccine recipients, 9 placebo recipients) who meet all the eligibility criteria will be included in the study. An over-enrolment of up to 10% (approximately 5 additional volunteers) will be accepted in the study.

5.2.3 Inclusion Criteria

1. Healthy, adult males and females;
2. Age at least 18 years on the day of screening and no greater than 60 years on the day of the first vaccination;
3. Available for follow up for the planned duration of the study (screening plus 18 months);
4. In the opinion of the principal investigator or designee, has understood the information provided. Written informed consent needs to be given before any study-related procedures are performed;
5. Willing to undergo HIV testing and counseling, and receive HIV test results;
6. If sexually active female, using an effective method of contraception (combined oral contraceptive pill; injectable contraceptive; diaphragm; cervical cap; intrauterine device; condoms; anatomical sterility in self or partner) from screening until at least 4 months after last vaccination, and willing to undergo urine pregnancy tests at time points indicated in the Schedule of Procedures (Appendix A);
7. If sexually active male, willing to use an effective method of contraception (such as condoms, anatomical sterility) from screening until 4 months after the last vaccination.

5.2.4 Exclusion Criteria

1. Clinically relevant abnormality on history or examination including history of immunodeficiency or use of systemic corticosteroids, immunosuppressive, antiviral, anticancer, or other medications considered significant by the designated trial physician in last 6 months;

2. Any acute or chronic medical condition requiring care of a physician (e.g.,, diabetes, coronary artery disease, rheumatologic illness, malignancy, substance abuse) that, in the opinion of the investigator, would preclude participation;

3. Any of the following abnormal laboratory parameters that are moderate, severe, or very severe: haematology (hemoglobin, absolute neutrophil count, absolute lymphocyte count, absolute CD4/CD8 count, platelets); urinalysis, biochemistries (total bilirubin, creatinine, AST, ALT). Volunteers with mild laboratory abnormalities that are judged by the principal investigator or designee to be not clinically significant may be enrolled. Refer to Appendix C for the grading of these laboratory parameters.

4. Reported high- risk behaviour for HIV infection, defined as:
   Within 6 months before vaccination, the volunteer has:
   - Had unprotected vaginal or anal sex with a known HIV infected person or a casual partner (i.e., no continuing established relationship)
   - Engaged in sex work for money or drugs
   - Used injection drugs (illicit), or
   - Acquired an STD;

5. If female, pregnant or planning a pregnancy within 4 months after last vaccination or lactating;

6. Receipt of blood transfusion or blood products 6 months prior to vaccination;

7. Receipt of a live attenuated vaccine (other than influenza) within 30 days or other vaccine within 14 days of vaccination;

8. Participation in another clinical trial of an investigational product currently or within past 12 weeks or expected participation during this study;

9. Receipt of another experimental HIV vaccine at any time;

10. Infected with HIV-1 or HIV-2 as indicated by ELISA and/or RT-PCR;

11. History of severe local or systemic reaction to vaccination or history of severe allergic reactions;

12. Confirmed diagnosis of hepatitis B (surface antigen, HbsAg); hepatitis C (HCV antibodies) or active syphillis;

13. History of grand-mal epilepsy, or currently taking anti-epileptics;

14. In the opinion of the investigator, unlikely to comply with protocol.

5.2.5 Recruitment of Study Volunteers

Healthy adult male and female volunteers will be recruited through various means including, but not limited to, the following: information presented in community organizations, hospitals, colleges, and other institutions, and/or advertisements to the general public. Interested parties may be given telephone contact information and may be invited to attend a seminar where basic information about the study is provided. Individuals who are interested in participating may have a telephone interview, which would let them know if they might qualify for study participation. They may be invited to attend additional informational seminars, at which detailed information about the study and the requirements for participation would be provided. Potential volunteers will be given a site-specific consent information sheet (sample Informed Consent document in Appendix B). They will have the opportunity to ask any questions they might have and to talk to a research team member. If they are still interested and willing to participate, they will be invited for a screening visit. Other processes, such as one-on-one sessions with study team members, may occur during which counseling will be provided and full informed consent will be obtained.

6.0 STUDY VISITS

6.1 Screening Visit

During the Screening Visit, site personnel will answer any questions about the study. Written site-specific informed consent will be obtained prior to conducting any study procedures. To ensure informed consent, the principal investigator or designee will discuss the following processes and explanations individually with each volunteer:

1) Pre HIV-test counseling
2) Risk-reduction counseling including safe-sex counseling
3) That it is unknown whether or not the study vaccines will protect against HIV infection or disease and, if so, the extent of that protection
4) That, following vaccination, it may be possible that the volunteer will develop antibodies against HIV, which may produce a positive reaction in a routine HIV test, and that provisions have been made to distinguish between response to vaccine and HIV infection during and after the trial.
5) That a sexually active volunteer should use a reliable form of contraception from screening, during the vaccination period until 4 months after the last vaccination.

A complete medical history and general physical examination (that includes weight, height, vital signs, examination of skin, respiratory, cardiovascular, central nervous and abdominal systems, as well as an assessment of cervical and axillary lymph nodes) will be performed. A pregnancy test will be performed for female volunteers and pre-test counseling will be conducted. Blood and urine specimens will be collected for all tests as indicated in the Schedule of Procedures (Appendix A). Laboratory test(s) may be repeated once at the discretion of the principal investigator or designee to investigate any isolated abnormalities.

If the screening visit occurs more than 42 days prior to date of vaccination, then study procedures for the screening visit must be repeated. However, the complete medical

history may be replaced by an interim medical history and the Informed Consent form may be reviewed.

6.2 Vaccination Visit Procedures

Site personnel will answer any questions about the study and review interim medical history and safety laboratory data to confirm eligibility. The Informed Consent form administered at the Screening Visit will be reviewed.

A directed physical examination and pre-HIV-test counseling will be performed. Blood and urine specimens will be collected. Pregnancy tests will be performed for female volunteers and results obtained prior to vaccination.

Vital signs (pulse, respiratory rate, blood pressure and temperature) will be recorded prior to vaccination. The site of injection and any systemic symptoms will be evaluated prior to vaccination and recorded. Volunteers will be closely observed for 30-45 minutes after each vaccination. Vital signs (pulse, respiratory rate, blood pressure and temperature) will be monitored at 30-45 minutes after each vaccination and recorded. Any local and systemic reactogenicity events, as well as any other event that occurs, will be recorded at 30-45 minutes.

For remaining vaccination visits, site personnel will answer any questions about the study, review interim medical history and collect safety laboratory samples. A directed physical examination will be performed that will include weight, vital signs, examination of injection site, as well as an assessment of axillary lymph nodes and any further examination indicated by history or observation. Pre-test counseling will be conducted if an HIV diagnostic test is required. Blood and urine specimens will be collected for all tests as indicated in the Schedule of Procedures (Appendix A).

6.3 Post-Vaccination Follow-up Visits

Volunteers will receive a phone call from the study staff between the 2nd and 4th day after each vaccination. They will be asked about any local and systemic reactogenicity or other adverse events. If they report a moderate or severe reactogenicity symptom or a moderate or severe adverse event, they will be asked to return to the study site. Volunteers at each dosage level will be asked to return to the study site 7 days after the first vaccination and between the 10th and 14th day after the second and third vaccination. Directed physical examinations will be done and local and systemic reactogenicity events will be assessed. In addition, blood and urine will be obtained for assessments specified in Schedule of Procedures (Appendix A).

In case of adverse event(s), the volunteer will be assessed and followed up by the clinical team. Supplemental visit(s) for further investigation can be planned at the discretion of the principal investigator or designee. Supplemental visit(s) may be recommended if clinically indicated or to clarify observations.

6.4 Additional Follow-up Visits

Assessments and procedures will be performed according to the Schedule of Procedures (Appendix A). Post HIV-test counseling will be performed as the HIV-test results become available.

6.5 Final Visit/Early Termination Visit

Assessments will be undertaken according to the Schedule of Procedures (Appendix A).

7.0 STUDY VACCINE AND PLACEBO

7.1 Description of Investigational Product

The ADVAX e/g + ADVAX p/n-t vaccine and placebo are manufactured under GMP conditions by Vical, Inc.

ADVAX e/g + ADVAX p/n-t contains Clade C HIV env and gag (eg) and Clade C HIV pol and nef/tat fusion (pnt) mixed in a 1:1 ratio.

The Placebo is 0.01M sodium phosphate, 150mM NaCl, pH 7.2.

The description of the investigational product are shown in Table 2 below.

Table 2
Description of Investigational Product

Vaccine/ Placebo	Dosage Level	Total Volume in Vial (ml)	Total Injected Volume (ml)	Route of Administration
ADVAX e/g +ADVAX p/n-t	0.2mg	1.2ml	1.0ml	IM
	1.0mg	1.2ml	1.0ml	IM
	4.0mg	1.2ml	1.0ml	IM
Placebo	NA	1.2ml	1.0ml	IM

IM=Intramuscular

7.2 Storage and Shipment of Investigational Product

Authorization to ship the investigational product to the site will be provided in writing by IAVI, upon confirmation that all required critical documents for shipment authorization are collected. The vaccines will be shipped to the respective sites on dry ice. The ADVAX e/g + ADVAX p/n-t vaccine and placebo are stored at -30°C +/- 10°C .

7.3 Dispensing and Handling of Investigational Product

The investigational product will be dispensed as specified by the sponsor. Designated site personnel will ensure that the allocation number on the vial matches the allocation number assigned to the volunteer.

The investigational product will be used as supplied by the manufacturers with no further preparation. Holding the vials in the hand thaws the investigational product. The vials should not be shaken. The required volume will be drawn into the appropriate syringe.

7.4 Administration of Investigational Product

Investigational product will be administered at time points specified in the Schedule of Procedures Appendix A).

The preferred site of first administration is the deltoid muscle of the non-dominant upper arm. It is suggested that alternating arms be used for each vaccination.

A 25-gauge needle between 1 and 1.5 inches long, and a 2.0 – 3.0 ml syringe will be used.

Complete instructions for the handling and administration of the investigational product are supplied in the Study Operations Manual.

7.5 Accountability and Disposal of Used and Unused Investigational Product

All used vials will be returned to the vaccine dispenser or pharmacy according to site-specific SOPs at the end of each vaccination visit. The date, allocation number and location of storage of the returned vials will be recorded on a log. During the trial, the product accountability form, the dispensing log and the log of returned vials will be monitored. At the end of the trial, the monitor will check all used and unused vials against the inventory before the vials are returned to the sponsor or destroyed on site upon written instructions from the sponsor.

8.0 STUDY PROCEDURES

8.1 Informed Consent

All volunteers will give their written consent to participate in the trial on the basis of appropriate information and with adequate time to consider this information and ask questions.

A volunteer's consent to participate must be obtained by having him/her sign and date an IRB-approved, site-specific informed consent form that is witnessed by a member of the trial team prior to initiation of study procedures. The signed and dated informed consent form will remain at the study site for verification. A copy of the signed and dated informed consent form will be offered to the volunteer. Those volunteers who decline to take a copy of the informed consent form will be required to indicate on the informed consent form that they declined.

Potential trial volunteers will be informed about the possible benefits and risks of the vaccine and that there may be unknown risks. The volunteer will have access to information about the procedures for obtaining medical treatment and/or compensation in the event of trial-related injuries resulting from their participation in this study. The volunteers will be made aware before consenting to participate that they are free to withdraw without obligation at any time and that such an action will not adversely affect any aspect of their social or legal benefits and they will not be denied access to available medical care for which they might otherwise be eligible. If any changes to the protocol are made that may affect the volunteer's decision to continue in the study, an amended informed consent will be provided to the volunteers. Volunteers will be notified if new safety information becomes available that may affect their willingness to continue in the study.

Family members, sexual partner(s) or spouse(s) will be offered education and counseling regarding a volunteer's participation in the trial *ONLY* with the written consent of the participating volunteer.

8.2 Medical History and Physical Examination

At the time of screening, a past medical history will be collected that will include details of any previous reaction to vaccination, history of epilepsy, and contraceptive practices. An Interim medical histories will be collected at time-points according to Schedule of Procedures (Appendix A).

A general physical examination will be conducted including weight, height, vital signs, examination of skin, respiratory, cardiovascular, central nervous and abdominal systems, as well as an assessment of cervical and axillary lymph nodes. At the time of vaccination and at select time-points thereafter, general and/or directed physical examinations will be performed according to Schedule of Procedures (Appendix A). A directed physical examination will include weight, vital signs, examination of injection site, as well as an assessment of axillary lymph nodes and any further examination indicated by history or observation.

8.3 HIV Testing and Counseling

Study personnel will assess volunteers for past and current risk of HIV infection and counsel them prior to collecting blood for an HIV test. Study personnel will perform post HIV-test counseling as indicated in the Schedule of Procedures (Appendix A). The counseling process will include information on HIV, safe sex practices and risk reduction. The objective of counseling is to ensure that volunteers have sufficient knowledge about HIV infection to understand for the purpose of the test, the implications of a positive or negative result and the standard of care available locally for HIV infection. Volunteers will be told that it is possible to have a false positive HIV test result due to a response to the vaccine. Additionally, risk reduction counseling, including safe-sex counseling, will be provided during the study to reinforce low-risk behavior.

8.4 Family Planning Counseling

Study personnel will counsel volunteers at screening and on the day of each vaccination about the importance of prevention of pregnancies and the use of condoms, as well as other effective family planning methods.

8.5 Blood Collection and Shipment

Venous blood will be collected at visits, usually from the antecubital fossa, according to the Schedule of Procedures (Appendix A). Up to 180ml will be collected at the first vaccination visit. Up to 140ml will be collected at other visits. At no time will the total volume of blood collected exceed 550ml over an 8 week period. All specimens will be handled according to site-specific SOPs and the IAVI Core Laboratory SOPs.

Frozen peripheral blood mononuclear cells (PBMC) will be shipped to the IAVI Core Laboratory, London, where ELISPOT and CFC assays will be performed. PBMC, plasma and serum samples will also be shipped to the Core Laboratory for repeat testing and quality control and for future assays related to HIV vaccine research and development (this is subject to approval by the Trial Steering Committee and IRB/IEC). Only a code number will identify the samples.

8.6 Reimbursement

Volunteers will be reimbursed to cover their travel expenses, as well as child care and time lost from gainful employment. Reimbursement will be outlined in the site-specific informed consent forms.

8.7 Randomization and Blinding

The randomization schedule will be prepared by the statisticians at the Data Coordinating Center (the EMMES Corporation). The randomization list will be sent to the vaccine manufacturer for labelling and packaging of study vaccine and placebo in a double blind fashion.

Study site staff and volunteers will be blinded only with respect to the allocation of placebo or vaccine to volunteers. Blinding will not apply to the assignment of dosage levels (low, middle or high dose). Volunteers will be informed about their group assignment once the data analysis is completed.

8.8 Unblinding Procedure

Unblinding of an individual volunteer is indicated only in the event of a medical emergency where the clinical management/medical treatment of the volunteer would be altered by knowledge of the group assignment.

A two-layered tear off sheet will be kept in a sealed envelope in a secure location at the site. In the unlikely circumstance that a volunteer's group assignment needs to be revealed (i.e., unblinded) to assist in the clinical management of a Serious Adverse Event, the principal investigator or designee (after consultation with the IAVI Medical Affairs Director) will tear off the slip from this sheet to reveal the code for that one individual. The reasons for unblinding should be documented and the EMMES Corporation should be notified. The tear off sheet must be returned to the EMMES Corporation at the end of the trial. Procedures and contact numbers for unblinding are outlined in the Study Operations Manual.

9.0 ASSESSMENTS

9.1 Safety Assessments

Data on local and systemic reactogenicity events as described below in this section will be solicited with specific questions. Data on other events will be collected with open-ended questions.

9.1.1 Local reactogenicity events

Local reactogenicity events will be collected prospectively by structured interviews on the vaccination and post-vaccination follow-up visits (see Sections 6.2, Vaccination Visit Procedures and 6.3 Post Vaccination Follow-up Visits); recorded and graded according to pre-established criteria (see Appendix C, Adverse Event Grading Toxicity Table).

Pain, tenderness and local reactogenicity events such as erythema/skin discoloration, edema, induration, formation of crust or scab, skin damage (vesiculation, ulceration) will be assessed and graded according to Appendix C, Adverse Event Grading Toxicity Table.

9.1.2 Systemic reactogenicity events

Systemic reactogenicity events will be collected prospectively by structured interviews on the vaccination and post-vaccination follow up visits (see Sections 6.2 and 6.3).

Vital signs (pulse, respiratory rate, blood pressure and temperature) will be measured prior to vaccination and 30-45 minutes post-vaccination by study personnel, graded according to Appendix C, Adverse Event Grading Toxicity Table, and recorded.

Feverishness, chills, headache, nausea, vomiting, malaise and myalgia, arthralgia and rash will be assessed prior to vaccination and 30-45 minutes post-vaccination, graded according to Appendix C, Adverse Event Grading Toxicity Table, and recorded. All medication required for treatment of adverse events will be recorded.

9.1.3 Other adverse events

Other adverse events will be recorded following an open question to volunteers, with the dates of commencement and resolution and any medication required. All adverse events will be followed to resolution. Serious Adverse Events will be collected during the entire study period. They will be graded as indicated in Appendix C, Adverse Event Grading Toxicity Table. For more information regarding adverse events, refer to Section 10.0, Adverse Events.

9.1.4 Routine laboratory parameters

As shown in Table 3 below, laboratory parameters will routinely include hematology, clinical chemistry, immunological assays, and urinalysis. Female volunteers will have urine β hCG assessed. The laboratory samples for these tests will be collected at the time points indicated in the Schedule of Procedures (Appendix A). In the event of an abnormal laboratory value, volunteers may be asked to have an additional sample collected at the discretion of the principal investigator or designee. Volunteers will be screened for syphilis and viral hepatitis (HBsAg and HCV antibodies) at the Screening Visit only.

Table 3
Laboratory Parameters

**Laboratory Parameter**	**Test**
Hematology	Full blood count, differential and platelet count
Clinical chemistry	Liver function tests: aspartate transferase (AST), alanine aminotransferase(ALT), total and direct bilirubin<br><br>Kidney function test: creatinine
Immunology:	CD4 and CD8 T cells (percentage and absolute count); anti-double stranded DNA
Urinalysis	Dipstick: protein, blood, glucose, ketones, esterase (leukocytes), nitrite. If abnormalities are found on dipstick analysis for protein, blood, or leucocytes, perform microscopy

UNCONTROLLED COPY

9.2 Immunogenicity Assessments

9.2.1 Antibody responses

Binding and neutralizing antibodies will be measured as specified in the Schedule of Procedures (Appendix A).

9.2.2 Cellular responses

Immunogenicity assays, including ELISPOT for monitoring the number of circulating T cells that can be stimulated to secrete cytokine and cytokine flow cytometry (as described in IAVI Core Lab SOPs), will be performed on samples using a.) peptide pools designed to determine the specific epitopes recognized and b.) peptides from different HIV-subtypes. Selected T cell responses may be further characterized for HLA restriction and epitope specificity.

9.3 Other Assessments

9.3.1 HLA typing

HLA typing will be done on samples collected at the first Vaccination Visit. The results of tissue typing will be kept confidential. Volunteers will not receive results of the HLA-typing.

9.3.2 HIV antibody test

Samples will be tested by standard ELISA according to the Schedule of Procedures (Appendix A).

9.3.4 Pregnancy test

A pregnancy test (to be conducted by a member of the study team) will be performed by measurement of Human Chorionic Gonadotrophin (β hCG) in urine samples collected from female volunteers of childbearing potential according to the Schedule of Procedures (Appendix A).

Results of pregnancy test must be obtained prior to each vaccination.

9.3.5 Concomitant medication

Volunteers will be asked about concomitant medication. Each medication taken during the course of the study and the reason for its use will be documented on the appropriate source document.

10.0 ADVERSE EVENTS

10.1 Definition

An adverse event (AE) is any untoward medical occurrence during the course of the study in a subject administered study product and that does not necessarily have a causal relationship with the study product. An AE can therefore be any unfavourable or

unintended sign (including an abnormal laboratory finding), symptom, or disease, temporally associated with the use of the study product whether or not related to the study products. (ICH E6, GCP 1.2)

Any adverse event prior to first vaccination will be reported as a pre-existing condition on the previous conditions source document.

10.2 Severity Grading of Adverse Events

Severity grading of all AEs will ultimately be the responsibility of the principal investigator or designee. Criteria for grading the severity of laboratory events and adverse events are listed in Appendix C, Adverse Event Grading Toxicity Table. Specific events are graded as mild, moderate, severe and very severe. The principle used to develop the grading criteria is shown in Table 4 below and the same principle should be used in grading any events that have not been anticipated in the Toxicity Table (Appendix C).

Table 4
Severity Assessment Criteria for AEs

	**Description of Severity**
Mild	Transient or mild discomfort (<48 hours) with some limitation of activity. No prescribed therapy needed, monitoring only.
Moderate	Mild to moderate impact on activity. May be able to work full-time; some assistance may be needed. May require minimal or no medical intervention; may only monitor.
Severe	Marked impact on activities of daily living. May work part-time with some assistance. Requires medical intervention.
Very Severe	Extreme limitation in activity, requires significant medical assistance.

10.3 Serious Adverse Events (SAEs)

An adverse event is reported as a “Serious Adverse Event” by ICH Good Clinical Practice (ICH GCP) criteria if it:

- **Results in death,**
- **Is life-threatening,** Note: The term “life-threatening” in the definition of “Serious” refers to an event in which the participant was at immediate risk of death. It does not include a reaction that, had it occurred in a more severe form, might have caused death.
- **Results in persistent or significant disability/incapacity,** Note: the term “persistent or significant disability/incapacity” in the definition of “Serious” refers to the substantial disruption of a person’s ability to conduct his/her normal life. Did the condition or event cause the participant to be permanently disabled, physically or mentally?
- **Requires in-patient hospitalization or prolongs existing hospitalization** Note: the term “hospitalization” in the definition of “Serious” refers to a

UNCONTROLLED COPY

condition or event that requires or prolongs inpatient hospitalization. It does not refer to a visit to the hospital emergency room. The participant must have been admitted to the hospital (usually includes an overnight stay). Any condition or event requiring a participant to be hospitalized qualifies as a Serious event, unless the participant is hospitalized for the following reasons:

- Surgery or procedure planned prior to the participant's entry into the trial, unless the timing of this intervention has been brought forward due a worsening of the original condition since the start of the trial.

- Elective (i.e., beneficial to the volunteer, but not essential for survival) treatment of an ongoing previous condition is NOT considered Serious. .

- Reasons other than an adverse event, for example, social factors such as a family can no longer look after a volunteer, it is too far to travel back and forth from home to the hospital for a series of treatments related to an ongoing previous condition, these should NOT be considered Serious Adverse Events

- **Is a congenital anomaly/birth defect** Note: the term "congenital anomaly/birth defect" in the definition of "Serious" refers to something different, abnormal, peculiar that exists at or dating from birth of a child born to the volunteer after vaccination.

- **Any other important medical condition** Note: Medical and scientific judgment should be exercised in deciding whether expedited reporting is appropriate in other situations, such as Important Medical Events that may not be immediately life-threatening or result in death or hospitalisation, but may jeopardize the participant or may require intervention to prevent one of the other outcomes listed in the definition above. These events should be considered Serious and it should be documented why such an event was considered to be an SAE. Examples are allergic bronchospasm requiring intensive emergency treatment, anaphylaxis, seizures or blood dyscrasias, autoimmune disease that did not result in hospitalisation or development of drug dependency.

10.4 Relationship to Study Product

The determination of the likelihood that the investigational product caused the (serious) adverse event will be provided by an investigator who is a qualified physician. The investigator must sign and date the source document that supports the causality.

The criteria below are intended as a guideline to assist the investigator in determining causality (not all criteria must be present):

Not Related: This category applies to those (S)AEs that, after careful medical consideration, are clearly felt to be due to extraneous causes (disease, environment, etc.) unrelated to the vaccine under investigation.

Unlikely: This category applies to those adverse events that, after careful medical consideration, are considered unlikely to be related to the trial vaccine with near certainty. In general, this category is applicable to an (S)AE that meets the following criteria:

1. It does not follow a reasonable temporal (time) sequence from administration of the vaccine.

2. It may have been readily produced by the volunteer's clinical state, environmental or toxic factors, or other modes of therapy administered to the volunteer.

3. It does not follow a known pattern of response to the vaccine.

Possibly: This category applies to those adverse experiences that, after careful medical consideration, are felt unlikely to be related to the vaccine although the possibility cannot be ruled out with certainty. An (S)AE may be considered possibly related if, or when:

1. It follows a reasonable temporal (time) sequence from administration of the vaccine.

2. It may have been produced by the volunteer's clinical state, environmental or toxic factors, or other modes of therapy administered to the volunteer.

3. It follows a known pattern of response to the vaccine.

Probably: This category applies to those adverse experiences that, after careful medical consideration, are felt with a high degree of certainty to be related to the trial vaccine. An (S)AE may be considered probably related, if:

1. It follows a reasonable temporal (time) sequence from administration of the vaccine.

2. It cannot be reasonably explained by the known characteristics of the volunteer's clinical state, environmental or toxic factors, or other modes of therapy administered to the volunteer.

3. It follows a known pattern or response to the suspected vaccine.

4. It reappears after administration of an additional dose of vaccine, if given.

Definitely: This category applies to those adverse experiences that, after careful medical consideration, are - with certainty - related to the trial vaccine.

10.5 Collection of adverse events

All adverse events will be collected from the time of first vaccination until 24 weeks after last vaccination. Serious adverse events will be collected for the duration of the study.

10.6 Reporting (Serious) Adverse Events

Non Serious Adverse Events:

Adverse events should be recorded on the appropriate source document and entered into the database as soon as possible.

Serious Adverse Events:

Serious Adverse Events (see Section 10.3) should be recorded on the IAVI SAE Reporting Form and reported to IAVI within 24 hours of the trial site becoming aware of the event. SAEs should be entered in the database as soon as possible.

Notification must be made by:

- email followed by telephone contact, or
- fax followed by telephone contact.

The minimum information required in reporting a SAE are the volunteer identification number, date of birth, gender, event description (in as much detail as is known at the time), onset date of event (if available), reason event is classified as serious, date of last vaccination, reporting source (name of principal investigator or designee), causality in investigator's opinion and name of investigator.

Serious Adverse Event reporting numbers and contacts are

e-mail:	SAE@iavi.org
SAE Telephone Hotline:	+ 1 212 847 1110
Fax Number:	+ 1 888 317 4215

The IAVI SAE Reporting Form should be completed with all the available information at the time of reporting. The principal investigator or designee is required to write a detailed written report with follow up to resolution of the SAE (i.e., the volunteer recovers or dies, or the condition becomes chronic but relatively stable).

Further details for reporting SAEs are outlined in the Study Operations Manual.

The principal investigator will notify the local IRB/IEC of SAEs according to their requirements. (Unexpected adverse events at the ADARC site that are moderate, severe or very severe should be reported to the Rockefeller IRB.) The sponsor will notify the Data and Safety Monitoring Board (DSMB) and the regulatory authorities.

When appropriate, the sponsor will also notify the regulatory authority and other trial site where the investigational product is being tested.

10.7 Clinical Management

Adverse events will be managed by the clinical trial team who will assess and treat the event as appropriate, including referral to an independent physician and/or department. If any treatment/medical care is required as a result of the harm caused by the study vaccine or study procedures, this will be provided free of charge.

10.8 Pregnancy

Although not considered an adverse event if a woman becomes pregnant during the study, it is the responsibility of the principal investigator or designee to report the pregnancy promptly using the same procedures for reporting SAEs. The pregnancy will be recorded on the appropriate source document. For follow up on a pregnancy refer to Section 12.1.1, Follow up After Discontinuation of Further Vaccination.

10.9 Intercurrent HIV Infection

Though not considered an SAE, intercurrent HIV infection in study volunteers must be reported promptly using the same procedures for reporting SAEs.

HIV infection cannot be caused by the vaccine. Volunteers will be tested for HIV-1 and HIV-2 antibodies, as indicated in the Schedule of Procedures (Appendix A). HIV testing at additional time points may be performed at the discretion of the volunteer and Investigator, as medical or social circumstances arise.

11.0 MANAGEMENT OF HIV ISSUES DURING AND FOLLOWING THE TRIAL

11.1 HIV Testing

Only volunteers without HIV infection at screening will participate in the study. All HIV screening tests and routine post-vaccination tests will be performed by an independent laboratory that will follow a predetermined protocol to distinguish immune response to the vaccine from HIV infection. Results will be reported to the sites as HIV-infected or HIV-uninfected to prevent unblinding of the volunteer and staff. If a volunteer is found to be HIV-infected, a newly drawn blood specimen will be collected for confirmation.

Should a volunteer require an HIV test outside the study for personal reasons, it is recommended that the volunteer contact the study staff first. The HIV test may be drawn at the clinical site and then processed at the independent laboratory as above. Written evidence of HIV status (infected or not infected) will be provided to the volunteer upon request.

11.2 HIV Infection

Volunteers who are found to be HIV infected at screening and study volunteers who acquire HIV infection during the trial will be managed in the following way:

11.2.1 Counseling

The volunteer will be counselled by the study counsellors. The counseling process will assist the volunteer with the following issues:

- Psychological and social implications of HIV infection

- Whom to inform and what to say
- Implications for sexual partners
- Avoidance of transmission to others in future

11.2.2 Referral for support and/or care

Volunteers will then be referred to a patient support centre or institution of his/her choice for a full discussion of the clinical aspects of HIV infection. Referral will be made to a designated physician or centre for discussion of options of treatment of HIV-infection.

11.3 Social Discrimination as a Result of an Antibody Response to Vaccine

The aim is to minimize the possibility of social discrimination in volunteers who develop vaccine-induced antibodies and therefore test positive on a routine ELISA test by providing appropriate diagnostic HIV testing and certification for volunteers as required, outlined above, both during and after the trial. In addition, a letter stating that the individual has participated in a vaccine trial, giving a contact number in case of medical emergency will be provided.

12.0 DISCONTINUATION OF VACCINATION AND/OR VOLUNTEER WITHDRAWAL FROM STUDY

12.1 Discontinuation of Further Vaccinations

Volunteers will be discontinued from further vaccination for any of the following reasons:

1. A disease or condition or an adverse event that may develop, regardless of relationship to the candidate vaccine, if the principal investigator or designee is of the opinion that further vaccination will jeopardize the safety of the volunteer.

2. An abnormal laboratory event based on the following criteria, defined in Section 10.6:

    - For a mild laboratory event, volunteers may be vaccinated only if the abnormality is judged to be not clinically significant by the principal investigator or designee,
    - For a moderate laboratory event, the laboratory test must be repeated and the event determined to be resolved in the opinion of the principal investigator or designee prior to vaccination,
    - For a severe or very severe laboratory event, even if resolved, the IAVI Medical Affairs Expert must be consulted before making a decision to vaccinate.

3. A severe local reactogenicity event involving the major circumference of the arm, not resolving within 72 hours.

4. Anaphylaxis; bronchospasm; laryngeal oedema; collapse; convulsions or encephalopathy.

5. Life threatening medical event following vaccination unless not related to the investigational product.

6. Any immediate hypersensitivity reaction judged due to investigational product.

7. Pregnancy.

8. Intercurrent HIV Infection.

9. Volunteer request to discontinue further vaccination.

Any volunteer discontinuing further vaccination or being considered for discontinuation of vaccine will be discussed with the IAVI Medical Affairs Expert.

12.1.1 Follow up after discontinuation of further vaccinations

Any adverse event resulting in the discontinuation of a volunteer's vaccinations will be followed up until resolution or until the adverse event is judged by the principal investigator or designee to have stabilized where possible. Immunological monitoring will continue, provided the volunteer is willing. The frequency of assessments will be determined by the Trial Steering Committee.

A pregnant volunteer will be followed until the end of the study and until delivery, if delivery occurs after the study has ended. Approximately 2-4 weeks after delivery, the baby will be examined by a pediatrician to assess the health status of the baby. The health status of the baby will be reported to IAVI.

Follow-up of HIV-infected individuals who have received investigational product will be determined by the Trial Steering Committee.

The date and reason for discontinuation of vaccination should be collected.

12.2 Withdrawal from the Study (Early Termination)

Volunteers may be withdrawn from the study permanently for the following reasons:

1. Volunteers may withdraw from the study at any time if they wish to do so, for any reason.
2. Following an adverse event at the discretion of the investigator (or designee) discretion.
3. The principal investigator or designee has reason to believe that the individual is not complying with the protocol.

12.2.1 Follow up after withdrawal from study (Early Termination)

Any adverse event resulting in the withdrawal of a volunteer will be followed up until resolution or until the adverse event is judged by the principal investigator or designee to have stabilized where possible.

At the time of the withdrawal, provided the volunteer is willing, all the requested termination visit procedures will be performed according to the Schedule of Procedures (Appendix A).

The date and reason for withdrawal from the study (early termination) should be collected. Volunteers who are withdrawn from the study (early termination) will not be replaced, but, wherever possible, will be followed up to the time of their final planned visit.

13.0 DATA HANDLING

13.1 Data Handling at the Trial Sites

Data will be collected by the clinical trial staff and entered on to the appropriate source document.

All source documents and laboratory reports will be reviewed by the clinical team and the data entry clerk, who will ensure that they are accurate and complete. Adverse events will be graded, assessed for severity and causality and reviewed by the principal investigator or designee as they arise.

Changes to source documents must be initialled and dated and the reason for change must be noted. All source documents will be kept in a secure location and held for 15 years. The written records for the trial will be held at the site.

13.2 Data Collection and Transfer at the IAVI Core Laboratory

Data generated at the IAVI Core Laboratory will be transferred directly to the Data Coordinating Center (the EMMES Corporation)

13.3 Data Entry at the Trial Sites

Data will be entered into the EMMES Internet-based Data Entry System (IDES). Consistency checks and range checks will be performed by data entry and supervisory personnel. Queries raised by the monitor or by The EMMES Corporation will be directed to the investigators and study staff. A file will be held at the clinic(s) for each local volunteer containing all the source documents and written documentation on all queries raised and how they were addressed.

13.4 Quality Assurance and Audit

To ensure the quality and reliability of the data gathered and the ethical conduct of this trial, standard operating procedures have been developed for all clinic and laboratory procedures. Regular monitoring and an independent audit of the trial will be performed according to Good Clinical Practice (GCP).

13.5 Data Handling and Analysis

The statistician at the Data Coordinating Center (EMMES Corporation), in collaboration with the principal investigators (or designees) and the sponsor, will create tables according to a data analysis plan that has been reviewed and agreed to by the principal investigators (or designees) and IAVI. The EMMES Corporation will conduct the data analysis and will provide interim and final study reports for the DSMB, IAVI, principal investigators (or designees) and the regulatory authorities as appropriate. Prior to an

analysis, additional monitoring visits will take place if necessary to validate the data held on the database , as well as all consent forms and dispensing records. Data files will be prepared by EMMES from a 'frozen' dataset for that particular analysis.

Unblinded data will be seen only by the DSMB prior to unblinding the trial. Preliminary grouped data analyses may be done without unblinding of volunteers, principal investigators, laboratory personnel, or IAVI to individual assignments.

13.6 Trial Supervision and Monitoring

The Data and Safety Monitoring Board (DSMB) and the Trial Steering Committee (TSC) will oversee the progress of the clinical trial.

The clinical study monitor will ensure that the trial is conducted, recorded and reported in accordance with the protocol, standard operating procedures, Good Clinical Practice (GCP), ICH Guidelines and the applicable regulatory requirements. The monitor will validate trial data held on the database against the source documents. The principal investigator, by signing the protocol, and the volunteers, by giving consent, agree that the monitor may consult and/or copy source records (clinical notes, laboratory values and other medical records if applicable) to accomplish this task. Such information will be treated as strictly confidential and will under no circumstances be made publicly available. The monitoring will adhere to Good Clinical Practice and ICH guidelines. Data that should be verifiable from source documents for trial volunteers include, but are not limited to:

- Documentation of any existing conditions or past conditions relevant to eligibility
- Signed consent forms
- Dates of visits including dates of vaccinations
- Reported laboratory results
- All adverse events
- Concomitant medications

14.0 STATISTICAL CONSIDERATIONS

14.1 Sample Size

The study uses a dose-escalation design of ADVAX e/g + ADVAX p/n-t vaccine candidate or placebo administered intramuscularly three times during the course of the study. The study investigates three dose levels of ADVAX e/g + ADVAX p/n-t vaccine, 0.2 mg, 1 mg, and 4 mg. Each dose group consists of 15 volunteers randomized in a 4:1 ratio of active vaccine to placebo.

A total of up to 45 volunteers will be enrolled in the study; 36 volunteers will be given active vaccines and 9 volunteers will be given placebo. The small sample size is appropriate for an exploratory dose-escalation study of a novel product while safety and tolerability and immunogenicity of the vaccine are investigated.

14.2 Statistical Power and Analysis

Vaccine Safety and Tolerability:

The rate of local and systemic reactogenicity events will be used to assess the differences between dose groups. The sample size of the study was restricted by safety

considerations. Nevertheless, the study has at least 80% power (at α=0.05 confidence level of an exact one-sided trend test) to detect the difference of 48 percentage points or more in the rate of events between the active (n_1=36) and the placebo (n_2=9) groups. The power of the statistical comparison was computed assuming 10% rate of events in the placebo group.

The rate of Serious Adverse Events related to vaccine will be used as one measure of the safety of the candidate vaccine. Adverse Events that may be temporarily incapacitating (for example, loss or cancellation of work or social activities), which could make a vaccine impractical for large scale use if they occur in more than a small proportion of cases, will also be assessed.

All adverse events will be reported, grouped as to whether or not they qualify as SAEs, their severity assessment, and their relationship to vaccine (as judged by the investigator and reviewed by the sponsor and the DSMB). The following example illustrates the limited statistical power of this initial Phase 1 study.

Prior to the enrollment of the medium dose level group:
If none of the volunteers receiving the vaccine experiences an SAE related to vaccine (n=12), the 95% upper confidence bound for the rate of these adverse events in the population is 0.22.

Prior to the enrollment of the highest dose level group:
If none of the volunteers receiving the vaccine experiences an SAE related to vaccine (n=24), the 95% upper confidence bound for the rate of these adverse events in the population is 0.12. Restricted to the medium dose level group (n=12), the upper confidence bound would be 0.22.

After completion of the study:
If none of the volunteers receiving the vaccine experiences an SAE related to vaccine (n=36), the 95% upper confidence bound for the rate of these adverse events in the population is 0.08. Restricted to the two highest dosage level groups (n=24), the upper confidence bound would be 0.12. Restricted to the highest dose level group (n=12), the upper confidence bound would be 0.22.

Vaccine Immunogenicity:

Cellular immune responses will be analyzed using binomial methods to examine for the presence or absence of HIV-specific T-cell responses quantified by ELISPOT and cytokine flow cytometry (CFC). Presence or absence of binding and neutralizing antibodies will be also analyzed. Assays will be performed in a similar fashion in all volunteers. Because of the small sample sizes and multiple epitopes, the results will be primarily descriptive. Nevertheless, the study has at least 80% power (at α=0.05 confidence level of an exact one-sided trend test) to detect the difference of 45 percentage points or more in the response rate between the active (n_1=36) and the placebo (n_2=9) groups. The power of the statistical comparison was computed assuming 5% response rate in the placebo group. The study has at least 80% power (at α=0.05 confidence level of an exact one-sided trend test) to detect the difference between the response rates of 20% and 72% in any two dose groups (n_1= n_2=12).

Based on the previous experience with IAVI Phase I vaccine studies, it is expected that the amount of missing, unused or spurious data will be insignificant. Unused and spurious data will be listed separately and excluded from the statistical analysis. Missing

data will be excluded from the statistical analysis. A data analysis plan will be developed and agreed upon by IAVI and the investigators prior to unblinding.

15.0 DATA AND BIOLOGICAL MATERIAL

All data and all biological material collected through the clinical trial shall be the joint property of the principal investigators or designees and IAVI.

The raw computerized data generated in this study will be held by the EMMES Corporation on behalf of IAVI and the principal investigators or designees. The clinical sites will also hold the frozen data files and tables generated for the purposes of analysis. Principal investigators or designees will have access to the EMMES database with appropriate blinding.

16.0 ADMINISTRATIVE STRUCTURE

The principal investigator will be responsible for all aspects of the trial at the study site.

16.1 Trial Steering Committee (TSC)

The supervision of the trial and the operational activities will be the responsibility of a Trial Steering Committee (TSC) consisting of the principal investigators and designees, and sponsor representatives. The TSC will meet regularly with representatives from the clinical, laboratory and data management teams.

16.2 Data and Safety Monitoring Board (DSMB)

The DSMB will consist of independent individuals who are not involved in the trial. No investigators responsible for the clinical care of trial volunteers or representative of IAVI may be a member of the DSMB. However, the DSMB may invite the principal investigators or designees and an IAVI representative to an open session of the meeting to provide information on study conduct, present data or to respond to questions at an open portion of the DSMB meeting.

The review of trial data by the DSMB will take place at least 14 days after the tenth volunteer in each dose group receives the second injection before proceeding to the next dose group. Additional meetings will take place if there are indications for an interim review, which may be unblinded at the discretion of the DSMB.

16.2.1 Content of interim review

The DSMB will be asked to review on an interim basis:

- All severe or very severe clinical adverse/reactogenicity events judged by the principal investigator or designee to be possibly, probably or definitely related to vaccine.
- All severe or very severe laboratory adverse events confirmed on retest and judged by the principal investigator or designee to be possibly, probably, or definitely related to vaccine.

16.2.2 Indications for discontinuation of vaccinations in all volunteers

If 3 or more of the volunteers participating in this trial develop an SAE judged definitely, probably or possibly related to the vaccine, the principal investigator or designee and IAVI will request a review by the DSMB and the trial will be suspended pending a review of all safety data by the DSMB which may be unblinded at the discretion of the DSMB. Following this review, the DSMB will make a recommendation to the principal investigators and IAVI regarding the continuation of the trial. Unless the trial is permanently discontinued, the staff, principal investigators/designees and IAVI will not be made aware of any unblinded results.

17.0 INDEMNITY

Trial and physician indemnity and product liability will be undertaken by IAVI, the trial sponsor. For research-related injuries and/or medical problems determined to result from receiving the study vaccine, treatment including necessary emergency treatment and proper follow-up care will be made available to the study volunteer free of charge.

18.0 PUBLICATION

A primary manuscript describing safety and immune responses in this trial will be prepared promptly after the data analysis is available, based on the data compiled by the IAVI statistical centre. Authors will be representatives of trial site, the statistical centre, the laboratories and IAVI, subject to the generally accepted criteria of contributions to the design, work, analysis and writing of the study. Manuscripts will be reviewed by representatives of each participating group.

19.0 ETHICAL CONSIDERATIONS

This study will be conducted in compliance with the protocol, ICH/GCP and applicable regulatory requirements. It will be reviewed and approved by the respective national authorities. The trial will not be initiated before the protocol and informed consent and volunteer information form have been reviewed and received approval/favourable opinion from the local IRB. Should a protocol amendment be made that needs IRB approval, the changes in the protocol will not be instituted until the amendment and revised informed consent (if appropriate) have been reviewed and received approval/favourable opinion from the local IRB. A protocol amendment intended to eliminate an apparent immediate hazard to volunteers may be implemented immediately providing the appropriate regulatory authorities and IRB are notified as soon as possible and an approval is requested. Protocol amendments for logistical or administrative changes only may be implemented immediately; the IRB need only to be informed.

The constitution of the IRB must meet the requirements of the participating country. A list of the IRB members with names and qualifications will be requested. If such a list is unavailable, the principal investigators or designees must provide the name and address of the IRB along with the statement from the IRB that it is organised according to GCP and the applicable laws and requirements of the participating country.

GLOSSARY

Abbreviation	Term
ALT	Alanine-Aminotransferase
anti-ds DNA	anti-double stranded DNA antibodies
AST	Aspartate-Aminotransferase
CFC	Cytokine Flow Cytometry
DSMB	Data and Safety Monitoring Board
DNA	Deoxyribonucleic Acid
ELISA	Enzyme Linked Immunosorbent Assay
GCP	Good Clinical Practice
HIV	Human Immunodeficiency Virus
IAVI	International AIDS Vaccine Initiative
ICH	International Conference on Harmonization
PMBC	Peripheral Blood Mononuclear Cells
SAE	Serious Adverse Event
TSC	Trial Steering Committee

REFERENCES

1. AIDS epidemic update December 2001. Joint UNAIDS/WHO, December 2001.

2. Beddows S, Lister S, Cheingsong R, Bruck C, Weber J. Comparison of the antibody repertoire generated in healthy volunteers following immunization with a monomeric recombinant gp120 construct derived from a CCR5/CXCR4-using human immunodeficiency virus type 1 isolate with sera from naturally infected individuals. J Virol 1999; 73:1740-1745.

3. Dolin, R. (2000). "HIV vaccines for prevention of infection and disease in humans." Infect Dis Clin North Am 14(4): 1001-16.

4. Graham, B. S. (2002). "Clinical trials of HIV vaccines*." Annu Rev Med 53: 207-21.

5. Goulder PJ, Rowland-Jones SL, McMichael AJ, Walker BD. Anti-HIV cellular immunity: recent advances towards vaccine design. AIDS 1999; 13 Suppl A:S121-S136.

6. Donnelly JJ, Ulmer JB, Shiver JW, Liu MA. DNA vaccines. Annu Rev Immunol 1997; 15:617-648.

7. Tighe H, Corr M, Roman M, Raz E. Gene vaccination: plasmid DNA is more than just a blueprint. Immunol Today 1998; 19:89-97.

8. Wang B, Merva M, Dang K, Ugen KE, Boyer J, Williams WV, et al. DNA inoculation induces protective in vivo immune responses against cellular challenge with HIV-1 antigen-expressing cells. AIDS Res Hum Retroviruses 1994; 10 Suppl 2:S35-S41.

9. Vogel FR, Sarver N. Nucleic acid vaccines. Clin Microbiol Rev 1995; 8:406-410.

10. McMichael AJ, Callan M, Appay V, Hanke T, Ogg G, Rowland-Jones S. The dynamics of the cellular immune response to HIV infection: implications for vaccination. Philos Trans R Soc Lond B Biol Sci 2000; 355:1007-1011.

11. McMichael AJ, Rowland-Jones SL. Cellular immune responses to HIV. Nature 2001; 410:980-987.

12. Tang DC, DeVit M, Johnston SA. Genetic immunization is a simple method for eliciting an immune response. Nature 1992; 356:152-154.

13. Liu MA, Yasutomi Y, Davies ME, Perry HC, Freed DC, Letvin NL, et al. Vaccination of mice and nonhuman primates using HIV-gene-containing DNA. Antibiot Chemother 1996; 48:100-104.Hanke T, McMichael AJ. Design and construction of an experimental HIV-1 vaccine for a year-2000 clinical trial in Kenya. Nat Med 2000; 6:951-955.

14. Wang B, Boyer J, Srikantan V, Ugen K, Gilbert L, Phan C, et al. Induction of humoral and cellular immune responses to the human immunodeficiency type 1 virus in non-human primates by in vivo DNA inoculation. Virology 1995; 211:102-112.

15. Boyer JD, Wang B, Ugen KE, Agadjanyan M, Javadian A, Frost P, et al. In vivo protective anti-HIV immune responses in non-human primates through DNA immunization. J Med Primatol 1996; 25:242-250.

16. Lu S, Manson K, Wyand M, Robinson HL. SIV DNA vaccine trial in macaques: post-challenge necropsy in vaccine and control groups. Vaccine 1997; 15:920-923.

17. Calarota S, Bratt G, Nordlund S, Hinkula J, Leandersson AC, Sandstrom E, et al. Cellular cytotoxic response induced by DNA vaccination in HIV-1-infected patients. Lancet 1998; 351:1320-1325.

18. MacGregor RR, Boyer JD, Ugen KE, Lacy KE, Gluckman SJ, Bagarazzi ML, et al. First human trial of a DNA-based vaccine for treatment of human immunodeficiency virus type 1 infection: safety and host response. J Infect Dis 1998; 178:92-100.

19. Center for Biologics Evaluation and Research, FDA, "Points to Consider on Plasmid DNA Vaccines for Preventive Infectious Disease Indications, " 22 December 1996, Docket No. 96N-0400.

20. Martinez-Salas E. Internal ribosome entry site biology and its use in expression vectors. *Curr Opin Biotechnol 1999* Oct;10(5):458-64

21. Schneider R, Campbell M, Nasioulas G, Felber BK, Pavlakis GN. Inactivation of the human immunodeficiency virus type 1 inhibitory elements allows Rev-independent expression of Gag and Gag/protease and particle formation. *J Virol 1997* Jul;71(7):4892-903

22. Kotsopoulou E, Kim VN, Kingsman AJ, Kingsman SM, Mitrophanous KA. A Rev-independent human immunodeficiency virus type 1 (HIV-1)-based vector that exploits a codon-optimized HIV-1 gag-pol gene. *J Virol 2000* May;74(10):4839-52

23. Haddad D, Liljeqvist S, Stahl S, Andersson I, Perlmann P, Berzins K, Ahlborg N. Comparative study of DNA-based immunization vectors: effect of secretion signals on the antibody responses in mice. *FEMS Immunol Med Microbiol 1997* Jul;18(3):193-202

24. Li Z, Howard A, Kelley C, Delogu G, Collins F, Morris S. Immunogenicity of DNA vaccines expressing tuberculosis proteins fused to tissue plasminogen activator signal sequences. *Infect Immun 1999* Sep;67(9):4780-6

25. Weiss R, Durnberger J, Mostbock S, Scheiblhofer S, Hartl A, Breitenbach M, Strasser P, Dorner F, Livey I, Crowe B, Thalhamer J. Improvement of the immune response against plasmid DNA encoding OspC of Borrelia by an ER-targeting leader sequence. *Vaccine 1999* Dec 10;18(9-10):815-24

26. Qiu JT, Liu B, Tian C, Pavlakis GN, Yu XF. Enhancement of primary and secondary cellular immune responses against human immunodeficiency virus type 1 gag by using DNA expression vectors that target Gag antigen to the secretory pathway. *J Virol 2000* Jul;74(13):5997-6005

27. Hanke, T., J. Schneider, et al. (1998). "DNA multi-CTL epitope vaccines for HIV and Plasmodium falciparum: immunogenicity in mice." Vaccine 16(4): 426-35.

28. Temin HM. Overview of biological effects of addition of DNA molecules to cells. J Med Virol 1990; 31:13-17.

29. Parker SE, Vahlsing HL, Serfilippi LM, Franklin CL, Doh SG, Gromkowski SH, et al. Cancer gene therapy using plasmid DNA: safety evaluation in rodents and non-human primates. Hum Gene Ther 1995; 6:575-590.

30. Mor G, Singla M, Steinberg AD, Hoffman SL, Okuda K, Klinman DM. Do DNA vaccines induce autoimmune disease? Hum Gene Ther 1997; 8:293-300.

31. Wolff JA et al. Long-term persistence of plasmid DNA and foreign gene expression in mouse muscle. Hum Molec Gen 1, 363-369. 1992.

32. Wang R, Doolan DL, Le TP, Hedstrom RC, Coonan KM, Charoenvit Y, et al. Induction of antigen-specific cytotoxic T lymphocytes in humans by a malaria DNA vaccine. Science 1998; 282:476-480.

33. Le TP, Coonan KM, Hedstrom RC, Charoenvit Y, Sedegah M, Epstein JE, et al. Safety, tolerability and humoral immune responses after intramuscular administration of a malaria DNA vaccine to healthy adult volunteers. Vaccine 2000; 18:1893-1901.

34. Boyer JD, Cohen AD, Vogt S, Schumann K, Nath B, Ahn L, et al. Vaccination of seronegative volunteers with a human immunodeficiency virus type 1 env/rev DNA vaccine induces antigen-specific proliferation and lymphocyte production of beta-chemokines. J Infect Dis 2000; 181:476-483.

35. Tellez I et al. HIV-1 specific T-cell responses in seronegative volunteers immunised with an HIV-1 gag-pol DNA vaccine. 2-2-2000. 7th Conference on retroviruses and Opportunistic Infections, San Francisco, California.

36.Cichutek Klaus: DNA vaccines: Development, Standardization and Regulation. Intervirology 2000;43:331-338. Dept. of Medical Biotechnology, Paul Ehrlich Institut, Langen, Germany

37. Kurth R. (Ann NY Acad Sci 1995;772:140-151)

38. Temin HM. Overview of Biological Effects of Addiction of DNA Molecules to Cells. Dev Biol Stand 1998; 93:37-44. McArdle Laboratory, University of Wisconsin, Madison, USA.

39. Parker SE et al: Plasmid DNA malaria vaccine: tissue distribution and safety studies in mice and rabbits. Hum Gene Ther 1999, 10:741-758.

40. Manam S. at al. Plasmid DNA Vaccines: Tissue Distribution and Effects of DNA Sequence, Adjuvants and delivery Method on Integration into Host DNA. Intervirology 2000; 43:273-281 (Merck)

41. Ledwith BJ et al: Plasmid DNA Vaccines: Investigation of Integration into Host Cellular DNA following Intramuscular Injection in mice. Intervirology 2000; 43: 258-272.

42. Martin T. et al: Plasmid DNA Malaria Vaccine: the potential for genomic Integration after Intramsucular Injection. Human Gene Therapy 10: 759-768 (March 20, 1999), supported by Vical.

43. Robertson JS, Griffiths E: Assuring the Quality, Safety and Efficacy of DNA Vaccines. Molecular Biotechnology Vol 17, 2001; 143-149

44.Smith HA, Klinman DM: The regulations of DNA vaccines. Current Opinion in Biotechnology; 2001; 12:299-303. Office of Vaccine Research and Review, Center for Biologics Evaluation and Research, Food and Drug Administration, Bethesda, MD

APPENDIX A: SCHEDULE OF PROCEDURES

[illegible]	[illegible]	[illegible]	[illegible]	[illegible]	[illegible]	[illegible]	[illegible]	[illegible]	[illegible]	[illegible]	[illegible]	[illegible]	[illegible]	[illegible]	[illegible]	[illegible]
Informed Consent	X															
ADVAX Vaccine/Placebo		X			X				X							
Medical History	X	X	X	X	X	X	X	X	X	X	X	X	X	X	X	X
General Physical Exam	X													X	X	X
Directed Physical Exam		X		X	X		X	X	X		X	X	X			
Pregnancy Test (women)	X	X			X				X				X		X	X
Pregnancy Avoidance Counseling	X	X			X				X			X				
Risk Reduction Counseling including Safe Sex Counseling		X			X			X	X			X	X	X	X	X
HIV Testing Pre-/Post HIV test Counseling[b]	X	X			X			X	X			X	X	X	X	X
Adverse Events		X	X	X	X	X	X	X	X	X	X	X	X	X		
Serious Adverse Events		X	X	X	X	X	X	X	X	X	X	X	X	X	X	X
Vital Signs (pre and 30-45 minute post vaccination)		X			X				X							
Local and Systemic Reactogenicity Assessment (pre- and post - vaccination		X	X	X	X	X	X		X	X	X					
Hep BsAg, anti-HepC, syphillis	X															
HLA Typing		X														
Hematology and Clinical Chemistry	X	X		X	X		X	X	X		X	X	X	X	X	X
Immunology (CD4/CD8)		X							X				X			X
Anti ds-DNA		X									X		X			X
Urinalysis	X	X		X	X		X		X		X			X	X	X
PBMCs for Cellular immunogenicity assays (ELISPOT assay and CFC) and storage		X		X			X				X		X	X	X	X
Binding Antibody Responses		X						X				X[c]				
Neutralizing Antibody Responses												X[d]				
Serum, plasma storage		X			X			X				X	X	X	X	X

[a] ET = Early Termination
[b] Post-test counseling will be offered after HIV test results are available, at the next study visit or when available according to site-specific SOPs.
[c] If strong responses are observed, additional samples may be run.
[d] If binding antibodies are detected, neutralizing antibodies will be assessed in volunteers in the highest dosage group at Week 16 only.

APPENDIX B: SAMPLE INFORMED CONSENT FORM

This appendix to be made site-specific and attached to this protocol by site

Consent Information Sheet for Screening and Participation in a Study with an Experimental HIV Vaccine

Protocol: IAVI C001

Title: A Randomized, Placebo-Controlled, Dose-Escalating, Double-Blinded Phase 1 Study to Evaluate the Safety and Immunogenicity of a Clade C DNA Vaccine (ADVAX) Administered Intramuscularly to HIV-Uninfected, Healthy Volunteers.

You have been asked to participate in the investigational research study named above. You are being asked to join this research study voluntarily as a healthy individual, not as an individual at high risk of developing human immunodeficiency virus (HIV).

You have the right to know about the procedures, risks, hazards, discomforts, and possible benefits of this study to help you make an informed decision about whether or not you will participate in the study. Please read the information below and ask questions about anything you don't understand before deciding whether or not to take part. This informed consent form does not replace any other informed consent forms you have signed.

Purpose of this study

Over 40 million people worldwide are currently infected with HIV, the virus that causes AIDS (Acquired Immune Deficiency Syndrome). The number of new cases continues to rise at an alarming rate. Other infectious diseases, such as smallpox or poliomyelitis, have been controlled, or even eliminated, by vaccination programs. Many experts believe that an HIV vaccine offers the best hope for controlling the epidemic.

Many different possible HIV vaccines are currently being developed and tested. This research study is a phase one study (first in humans) to determine whether this candidate (experimental) HIV vaccine is safe for use. The study will also look at how your immune system responds to the vaccine. This trial is one stage of testing this vaccine in humans; further studies will have to be carried out to determine whether this vaccine protects the recipient from getting HIV or AIDS before the vaccine can be used in the general population.

Background

The vaccine was designed by scientists at the Aaron Diamond AIDS Research Center (ADARC) and was manufactured at Vical, Inc. The study sponsor is the International AIDS Vaccine Initiative (IAVI), an international, scientific, non-profit organization, whose mission it is to develop a safe and effective preventive vaccine against HIV, the virus that causes AIDS. This project is one of several vaccine development programs of IAVI.

Selection of study participants
This study will enroll approximately 45 healthy HIV-1 and HIV-2 negative male or female volunteers at lower risk of HIV infection, aged between 18-60 years, who are well informed about the study and provide written informed consent.

Duration of the study
The entire study will last approximately 19 months, including screening.

Study procedures
Pre-screening
- Before screening, you will have the opportunity to obtain basic information about the details of the study. You will have the opportunity to talk to the study doctors and nurses and ask them questions.

Screening
- Screening will determine whether or not you are eligible for the study.
- If you agree to be screened, you will sign 2 copies of the Informed Consent Form confirming that you have been informed about the study and voluntarily agree to take part. One copy is for you to keep and one will be kept in your confidential study file. If you do not wish to keep your copy, you will sign a form that states you have declined to take your copy and we will keep it for you.
- You will be asked questions about your general health and your sexual behavior.
- Pre-HIV test counseling will be provided.
- Up to 60 mL (about 4 tablespoons) of blood will be drawn to test for HIV and other health conditions.
- A urine specimen will be collected for testing, including a pregnancy test for females with childbearing potential.
- The screening visit will take about 1 hour.

Your participation at screening is entirely voluntary. Attending information seminars and undergoing prescreening or screening tests do not obligate you to continue participation in this vaccine study.

Study Participation
If you are found eligible through the screening process, you have the option of participating in the study.
- You will have another opportunity to talk to and ask questions of the study doctors and nurses. The Informed Consent Information Sheet will be reviewed with you again to ensure that you have been fully informed about the study.
- You need to know that:
  - Your participation is voluntary, that is, it is entirely up to you whether you choose to participate in this study or not.
  - Attending information seminars and undergoing prescreening or screening tests do not obligate you to join the vaccine study.
  - If you decide not to participate, none of your legal rights will be compromised.
  - You may withdraw your consent to participate at any time, for any reason, without prejudice.
  - If you decide not to participate in this research study, there might be another HIV vaccine trial in the future. Currently, there is no licensed HIV vaccine available.

- If you decide to participate in the study:
  - You will be assigned a study identifier.
  - On the day of the study vaccination visit you will need to be at the clinic for approximately 1-1/2 hours. A medical history, physical exam and blood and urine will be collected for testing prior to giving you the study vaccine.
  - You will receive three vaccinations given in alternating upper arms. The vaccinations will be given at the initial study visit following the screening visit, and then 4 weeks and 12 weeks later.
  - In addition to the vaccination visits, you will have 3 phone calls (2-4 days after each vaccination) and 10 scheduled visits to the clinic for medical evaluations spread over a period of 78 weeks (18 months). These follow-up visits will last about 30 minutes each.
  - Approximately 140 ml (less than 10 tablespoons) of blood will be taken at 3 of your study visits. Approximately 120 ml (about 8 tablespoons) will be taken at 5 of your study visits. Approximately 20 ml (less than 2 tablespoons) of blood will be taken at one of your study visits. Approximately 10 ml (less than 1 tablespoon) will be taken at one of your study visits.
  - Females of childbearing potential will have a urine pregnancy test prior to receiving each vaccine dose. This test is being done because the safety of the vaccine for pregnant women and their fetuses is not yet known. There will also be pregnancy tests at the Week 28, Week 52 and Week 78 study visits . If you become pregnant during the study, you will be followed up until the delivery of the baby, and the baby will be examined by a pediatrician approximately 2-4 weeks after birth.

Study Vaccine
The vaccine being tested in this study is artificially made. It does not contain any material from live HIV. It does not contain blood or blood products, or material from individuals who are infected with HIV and does not contain material from individuals who have been found to be resistant to HIV. The vaccine contains artificially made DNA (genetic information) that resembles a small part of the DNA found in HIV. This may create a response from your immune system. Placebo is an inactive salt-water solution that looks like the active study vaccine.

It is absolutely NOT POSSIBLE to get HIV infection from this vaccine.

Study Design
This study is called a dose-escalation study. The dose of the vaccine will be incrementally increased after the data collected on the volunteers is reviewed to ensure that it is safe to continue with the study.

Volunteers will be screened for the study on a first-come, first-served basis. If you qualify for the study and wish to join, you will then be assigned randomly by a computer (which means by chance or like in a lottery) to receive either study vaccine or placebo. There is a four out of five chance that you will receive the study vaccine and a one out of five chance that you will receive the placebo. A placebo looks like the vaccine, but it does not contain the vaccine. All volunteers will receive three injections. There will be 3 groups in the study. Each group will have 15 volunteers.

- Group 1: 12 volunteers will receive the lowest dose of the vaccine. 3 volunteers will receive the placebo.

- Group 2: 12 volunteers will receive the middle dose of the vaccine. 3 volunteers will receive the placebo.
- Group 3: 12 volunteers will receive the highest dose of the vaccine. 3 volunteers will receive the placebo.

The information as to which group (vaccine or placebo) you are assigned to (the code) is kept secret in a sealed envelope. Only in the case of a serious medical event may the code be broken by a principal investigator after consultation and agreement with other physicians and scientists on the study team.

The investigators and volunteers will not know whether a volunteer has received vaccine or placebo until the end of the study. Only the people who generate the code list, the people who package and label the vaccines and possibly the Data and Safety Monitoring Board will know the code. None of these people will be involved in any clinical or laboratory evaluations during the trial.

Storage of Blood
After completion of the initial laboratory tests on your blood specimens, some of the remaining blood will be stored. A unique study identifier rather than your name will be used to label this blood. The stored samples may be used in the future for follow-up safety testing, quality control purposes and other tests related to approved HIV vaccine research and development, and may therefore be shipped to independent national or international laboratories.

Genetic Testing
A test will be conducted to determine your HLA type. HLA is a specific characteristic on the surface of your body cells. This information will help the researchers to understand differences in immune responses to the vaccine. The result of this test will be kept strictly confidential.

Risks and Discomforts
Blood tests for screening and study visits will be done by inserting a needle into your vein and can cause temporary local pain, bruising, and, rarely, infection. Following administration of the vaccine, you may experience pain, soreness, redness/discoloration and bruising around the site of the injection. With any new medicine or vaccine, there is a possibility of totally unexpected side effects, although previous testing indicates that this is unlikely.

This is the first study to test this vaccine in people. However, the vaccine has been shown to be safe in rabbits. People have received similar HIV vaccines made from DNA, which were safe and well-tolerated in the people who received them.

This study focuses primarily on how well the vaccine is tolerated (vaccine safety) and is not looking to see if the vaccine can prevent HIV infection or disease. Until larger studies have been performed, we will not know whether this vaccine is effective in protecting you from getting HIV or AIDS or slowing HIV disease. Therefore you should continue to avoid any behavior that may put you at risk of contracting HIV. **YOU SHOULD NOT CONSIDER YOURSELF PROTECTED FROM HIV AFTER PARTICIPATING IN THIS STUDY.**

We do not know what effect the vaccine would have on an unborn child if given to a pregnant woman. Females of child-bearing potential should use a reliable form of contraception, as discussed with a nurse counselor, until 4 months after the last vaccination. A pregnancy test will be carried out at screening, prior to the

vaccinations, at the Week 28 and 52 visits, and at study completion. Males who are not anatomically sterile should use condoms for 4 months after receiving the vaccination to avoid pregnancy in a spouse or partner.

Following vaccination you may test HIV positive on a routine HIV test. This does not mean that you have HIV or AIDS. It could mean that your body has been exposed to the vaccine and has produced antibodies to it. In case of a positive HIV test result, an independent laboratory will confirm whether you test positive as a result of the vaccination or whether you became infected with HIV through exposure in the community.

Should you get naturally infected with HIV due to exposure in the community, you will be referred for support and care that is standard.

Should you require an HIV test outside the study for whatever reason, we strongly recommend you contact the study team first. We will offer HIV testing at an independent laboratory that can distinguish between a positive result from a vaccine and a true HIV infection. To help avoid any problems, you will be offered an identification card that shows that you have joined this study. A contact number in case of queries or medical emergencies will be provided.

It is unknown whether receiving this HIV vaccine will alter your immune response to any future HIV vaccine that you might receive.

You should not donate blood while you are participating in the study.

Benefits

There are no direct benefits for you in taking part in the study except that you will get information about your general health and your HIV status, and you will receive HIV counseling. However, the information that we gain from this study may help to develop an effective HIV vaccine, which would benefit others.

Injuries

We do not expect you to suffer any injury as a result of participating in this study. However, in case you are injured as a result of being in this study, you will be given the necessary treatment for your injuries without charge. This treatment will include any necessary emergency treatment and appropriate referral, as needed. If you have any symptoms or medical problems that you think are due to getting this vaccine, you should report them right away to ________________________at telephone number ________________.

Circumstances for discontinuation or withdrawal from the trial

Your participation in the trial is completely voluntary. You can withdraw from the study at any time without giving a reason. Withdrawal will NOT compromise any rights you may have or influence any current or future medical care you may need.

You may be removed from the study without your consent for the following reasons:

- If your doctor feels that staying in the study is harmful to your health
- If you don't keep appointments
- If you have serious side effects from the vaccination
- If the Data and Safety Monitoring Board feels that the study should be stopped
- If the study sponsor decides to stop or cancel the study for any reason

New information
You will be told about any new information gained during the course of the study that might cause you to change your mind about staying in the study. You will be told about findings in this trial, and trials elsewhere, regarding the safety or effectiveness of this vaccine , as well as other HIV vaccines. You will also be told if a safe and effective HIV vaccine becomes available.

Supervision of the study
The trial will be supervised by a Trial Steering Committee. All data collected will be regularly checked by independent monitors and an independent Data and Safety Monitoring Board.

Confidentiality
Your participation in the study will be kept strictly confidential. You will be identified by a unique identity number known only to you and the clinic staff. All information collected about you , as well as all results of laboratory tests will be marked by this number and kept strictly confidential. In addition to the study staff that you meet, there may be other staff from national or international government regulatory agencies, members of the Data and Safety Monitoring Board, monitors, auditors, inspectors, and representatives of the sponsor who will check the records to make sure that the trial was conducted properly. They are equally bound to respect your confidentiality. Your identity will not be disclosed in any publication or presentation of this study.

End of Study
An analysis of the results will be performed after all volunteers have completed the study. This analysis will take about 6 to 12 months to complete. You will be told whether you received the study vaccine or the placebo.

Reimbursement
You will be reimbursed for the costs of travel, time, and child care ($100) for each study visit.

Contact numbers
If you have any further concerns or questions about the study or during the study, please feel free to call and discuss them with us.

If you have any questions regarding the study or your participation in the study, you may call____.

If you would like to discuss your rights as a research subject or aspects concerning your participating in the study, you may contact

________________________________.

INFORMED CONSENT FORM

IAVI C001

I, (name of subject) ....

of (address)
....

agree to take part in the research project entitled:

A Randomized, Placebo-Controlled, Dose-Escalating, Double-Blinded Phase 1 Study to Evaluate the Safety and Immunogenicity of a Clade C DNA Vaccine (ADVAX) Administered Intramuscularly to HIV-Uninfected, Healthy Volunteers.

I confirm that the nature and demands of the research have been explained to me and I understand and accept them. I understand that my consent is entirely voluntary and that I may withdraw from the research project if I find that I am unable to continue for any reason and this will not affect the legal rights I may otherwise have.

Participant:
Print Name: .... Signature: ....

Date: |__|__|/|__|__|__|/|__|__|__|__| Time: |__|__|: |__|__| (24 hours)
D D M M M/ Y Y Y Y

Person obtaining consent:
I have explained the nature, demands and foreseeable risks of the above research to the subject:

Print Name: .... Signature ....

Date: |__|__|/|__|__|__|/|__|__|__|__| Time: |__|__|: |__|__| (24 hours)
D D M M M/ Y Y Y Y

Principal Investigator or designee:

Print Name:.......................... Signature: ....

Date: |__|__|/|__|__|__|/|__|__|__|__| Time: |__|__|: |__|__| (24 hours)
D D M M M/ Y Y Y Y

Consent Copies

_____☐ I have been provided a copy of this signed and dated consent.
initials

_____☐ I have declined my copy of this signed and dated consent.
initials

APPENDIX C: ADVERSE EVENT GRADING TOXICITY TABLE

Abbreviations:

ADL	Activities of Daily Live	NA	Not applicable
FEV1	Forced expiratory volume in 1 second	OTC	Over the Counter
ICU	Intensive Care Unit	Rx	Treatment/Therapy
LLN	Lower Limit of Normal	ULN	Upper Limit of Normal
NSAID	Non-steroidal anti-inflammatory drugs	TEN	Toxic Epidermal Necrolysis

1. EXPECTED VACCINATION REACTIONS (PROSPECTIVELY COLLECTED)				
	**SEVERITY GRADING**			
**PARAMETER**	**MILD**	**MODERATE**	**SEVERE**	**VERY SEVERE**
**1.1. LOCAL REACTOGENICITY EVENTS**				
Local Pain (pain without area being touched)<br>Tenderness (pain when area is touched)	Minimal discomfort associated with minimal or no limitation of use of limb	Notable discomfort with limitation of use of limb causing greater than minimal interference with ADLs<br>**AND/OR**<br>One dose of a analgesic or NSAID medication required for this event	Significant discomfort with symptoms causing significant incapacity*<br>**AND/OR**<br>If taking analgesics or NSAID, repeated doses required	Hospitalisation due to these symptoms
Erythema or<br>Skin discoloration	Light red blush or mild discoloration up to a quarter of the circumference of the upper arm	Marked redness or discoloration with or without oedema involving up to 50% of the circumference of the arm	Brick red or significant discoloration with secondary infection or phlebitis involving the major part of the circumference of the upper arm	Necrosis
Edema (swelling)	Mild edema involving up to 25% of the circumference of the upper arm	Moderate edema involving up to 50% of the circumference of the arm	Marked edema involving between 50 and 75% of the circumference of the upper arm	Severe edema involving greater than 75% of the circumference of the upper arm
Skin damage (Vesiculation, Ulceration)	Vesicles or superficial disruption of epithelium < 1 cm	Vesicles or superficial disruption of epithelium < 2 cm	Full thickness disruption of the epithelium (ulceration) < 2 cm<br>**AND/OR**<br>not healing within 2 weeks	Necrosis
Induration (size)	Diameter < 1.5cm<br>**OR**<br>Duration < 2 weeks	Diameter 1.5-3 cm<br>**OR**<br>Duration 2-4 weeks	Diameter > 3 cm<br>**OR**<br>Duration > 4 weeks	Secondary infection or ulceration

UNCONTROLLED COPY

Formation of crust or scab	Ccrust or scab ≤ 2cm	Crust or scab > 2cm	Crust or scab > 4cm	Crust or scab > 6cm
**1.2. SYSTEMIC REACTOGENITY EVENTS**				
Chills	Minimal or no interference with ADL	Symptoms causing greater than minimal interference with ADL<br>**AND/OR**<br>Requires > 24 hours of OTC medication	Marked symptoms causing significant incapacity*	NA
Malaise	Minimal or no interference with ADL	Notable symptoms causing greater than minimal interference with ADL	Marked symptoms causing significant incapacity*	NA
Myalgia	Symptoms at other than injection site, but causing minimal or no interference with ADL	Notable muscle tenderness at other than injection site causing greater than minimal interference with ADL<br>**AND/OR**<br>One dose of an analgesic or NSAID medication required for this event	Symptoms causing significant incapacity*<br>**AND/OR**<br>If taking analgesic or NSAID, repeated doses required	Frank myonecrosis
Headache	Noticeable pain but causing minimal or no interference with ADL	Notable pain causing greater than minimal interference with ADL<br>**AND/OR**<br>One dose of an analgesic or NSAID medication required for this event	Intractable and causing significant incapacity*<br>**AND/OR**<br>If taking analgesic or NSAID repeated doses required	Requiring hospitalisation (other than emergency room visit)
Fever	37.7 – 38.6°C<br>99.8 – 101.5° F	38.7 – 39.3°C<br>101.6 – 102.7° F	39.4 – 40.5°C<br>>102.8 – 104.9° F	> 40.5°C<br>> 105° F
Nausea	Transient (< 24 hours) or intermittent symptoms resulting in minimal or no interference with intake	Persistent symptoms resulting in decreased intake for 24-48 hours	Minimal intake for > 48 hours	Hypotensive shock
Vomiting	Transient or intermittent vomiting resulting in minimal or no interference with ADL	Persistent episodes of vomiting resulting in interference with ADL	Associated with vomiting of all food/fluids in 24 hours and orthostatic hypotension requiring IV fluid/therapy	Hypotensive shock

UNCONTROLLED COPY

2. OTHER SYSTEMIC EVENTS

	SEVERITY GRADING			
**PARAMETER**	**MILD**	**MODERATE**	**SEVERE**	**VERY SEVERE**
Fatigue	Minimal or no interference with ADL	Notable symptoms causing greater than minimal interference with ADL	Marked symptoms causing significant incapacity*	NA
Allergic Reaction	NA	Localized urticaria at injection site	Generalized (beyond injection site) urticaria	Laryngospasm, bronchospasm, angioedema, or anaphylaxis
Arthralgia<br>*(See also Arthritis)*	Noticeable pain, but causing minimal or no interference with ADL	Notable pain causing greater than minimal interference with ADL<br>**AND/OR**<br>One dose of a analgesic or NSAID medication required for this event	Symptom causing significant incapacity*<br>**AND/OR**<br>If taking analgesics or NSAID, repeated doses required	Hospitalisation for this diagnosis
Pruritis	Localized and relieved spontaneously or with < 24 hours of OTC treatment (topical or systemic)	Itching beyond the injection site<br>**OR**<br>Localized requiring > 24 hours of OTC treatment (topical or systemic)	Generalized (beyond injection site) and poorly controlled despite treatment measures, seeks medical attention, and requires additional systemic treatment	Progressive symptoms
Rash/Dermatitis<br>*(See also Pruritis, if applicable)*	Erythematous, non-urticarial lesions	Diffuse rash of maculopapular lesions or dry desquamation	Vesiculation<br>**AND/OR**<br>Ulceration<br>**AND/OR**<br>Moist desquamation	ANY ONE: mucous membrane involvement, suspected Stevens-Johnson (TEN), erythema multiforme, necrosis requiring surgery, or exfoliative dermatitis

3. LABORATORY PARAMETERS (ROUTINELY COLLECTED)

HEMATOLOGY (mm^3 is equivalent to μl)

Haemoglobin	10.0 g/dL – 11.0 g/dL<br>**OR**<br>any decrease ≥ 2.5 g/dL	9.0 g/dL – 9.9 g/dL<br>**OR**<br>any decrease ≥ 3.5 g/dL	7.0 g/dL – 8.9 g/dL<br>**OR**<br>any decrease ≥ 4.5 g/dL	< 7.0 g/dL
WBC—Elevated	13,000 – 14,999/mm^3	15,000 – 19,999/mm^3	20,000 – 24,999/mm^3	>= 25,000/mm^3
WBC—Decreased	2000 – 2499/mm^3	1500 – 1999/mm^3	1000 – 1499/mm^3	< 1000/mm^3

UNCONTROLLED COPY

	SEVERITY GRADING			
**PARAMETER**	**MILD**	**MODERATE**	**SEVERE**	**VERY SEVERE**
Absolute Neutrophil Count	1000 – 1300/mm^3	750 – 999/mm^3	500 – 749/mm^3	< 500/mm^3
Absolute Lymphocyte Count	600 – 649/mm^3	500 – 599/mm^3	350 – 499/mm^3	< 350/mm^3
Absolute CD4 Count (HIV Negative)	300 – 400/mm^3	200 – 299/mm^3	100 – 199/mm^3	< 100/mm^3
Platelets—Decreased	100,000 – 124,999/mm^3	50,000 – 99,999/mm^3	25,000 – 49,999/mm^3	< 25,000/mm^3
Platelets—Elevated	NA	550,000 – 600,000/mm^3	> 600,000/mm^3	NA
**CHEMISTRIES**	ULN = Upper Limits of Normal			
BILIRUBIN (total) Hyperbilirubinemia	1.0 – 1.5 x ULN	>1.5 – 2.5 x ULN	>2.5 – 5.0 x ULN	> 5.0 x ULN
CREATININE	1.1 – 1.3 x ULN	>1.3 – 1.8 x ULN	>1.8 – 2.5 x ULN	> 2.5 x ULN
LIVER FUNCTION TESTS (LFTs)				
AST (SGOT)	1.51 – 3.0 x ULN	>3.0 – 6.0 x ULN	>6.0 – 10.0 x ULN	> 10.0 x ULN
ALT (SGPT)	1.51 – 3.0 x ULN	>3.0 – 6.0 x ULN	>6.0 – 10.0 x ULN	> 10.0 x ULN
**URINALYSIS**				
PROTEINURIA	1 + (30 mg/dl)	2 - 3 + (100-500mg/dl)	4 + (> 500mg/dl)	----
Random urine 24-hour urine	200 mg – 500 mg loss/day	> 500 mg – 1.0 g loss/day	> 1.0 – 3.0 g loss/day	Nephrotic syndrome **OR** > 3.0 g loss/day
HEMATURIA (in the absence of vaginal bleeding) By microscopic exam only	1+ (ca. 5 – 10 Ery/µl) 6-10 Ery/hpf	2+ (ca. 10-25 Ery/µl) >10 Ery/hpf	3+ (ca. 50 Ery/µl) **OR** Gross, with or without clots **OR** RBC casts	4+ (ca. 250 Ery/µl) Transfusion required
GLUCOSURIA	1+ (30mg/dl, 2.8 mmol/l)	2+ (100mg/dl, 5.5mmol/l)	3+ (300mg/dl, 17 mmol/l)	4+ (1000mg/dl, 55 mmol/l)
LEUCOCYTURIA	1+ (ca. 10-25 Leuco/µl)	2+ (ca. 75 Leuco/µl)	3+ (ca. 500/µl)	

4. OTHER CLINICAL OBSERVATIONS				
	SEVERITY GRADING			
**PARAMETER**	**MILD**	**MODERATE**	**SEVERE**	**VERY SEVERE**
**CARDIOVASCULAR**				
Hypotension	Transient orthostatic hypotension with heart rate increased > 20 beats/min<br>**OR**<br>Decreased by >10mm Hg systolic BP, No Rx required	Symptoms<br>**OR**<br>BP decreased by >20 mm Hg systolic, correctable with oral fluid Rx	IV fluid required<br>**OR**<br>Hospitalisation	Mean arterial pressure < 60 mm Hg<br>OR<br>End organ damage<br>OR<br>Shock, vasopressor Rx required
Hypertension	NA	> 150/100 mmHg (either or both values)	> 170/110 mmHg (either or both values)	Malignant hypertension
Cardiac Arrhythmia	Asymptomatic with transient dysrhythmia causing no interference with ADL	Notable symptoms causing interference with ADL	Causing significant incapacity*	Unstable dysrhythmia requiring hospitalisation and treatment
Pericarditis	NA	Minimal asymptomatic effusion requiring no treatment	Symptomatic effusion	Tamponade<br>**OR**<br>Pericardiocentesis/ surgery required
Haemorrhage, blood loss	Asymptomatic and requiring no therapy	Mildly symptomatic	Gross blood loss<br>**AND/OR**<br>1 – 2 units transfused	Massive blood loss<br>**AND/OR**<br>> 2 units transfused
**GASTROINTESTINAL**				
Constipation	NA	Significant abdominal pain with impaction requiring prescription	Requiring disimpaction<br>**AND/OR**<br>Hospital treatment	Distension with vomiting<br>**AND/OR**<br>Obstipation
Diarrhoea	Transient or intermittent episodes of unformed stools resulting in minimal or no interference with ADL	Persistent episodes of unformed-to-watery stools resulting in greater than minimal interference with ADL	Orthostatic hypotension requiring IV fluid/therapy<br>**AND/OR**<br>Bloody diarrhoea	Hypotensive shock
Oral Discomfort/Dysphagia	Mild discomfort, no difficulty swallowing	Difficulty swallowing but able to eat and drink	Unable to swallow solids	Unable to drink fluids; IV fluids required

	SEVERITY GRADING			
**PARAMETER**	**MILD**	**MODERATE**	**SEVERE**	**VERY SEVERE**
**NEUROLOGIC**				
Neuro-psych/mood	NA	Depression or anxiety symptoms causing individual to seek attention and be treated with counseling and/or pharmacotherapy	Severe mood changes requiring additional medical intervention<br>**AND/OR**<br>Suicidal ideation/gesture	Suicidal attempt
Paresthesia (burning, tingling, etc.)	Minimal discomfort resulting in minimal or no interference with ADL	Notable symptoms resulting in greater than minimal changes in ADL	Marked and persistent discomfort resulting in significant incapacity*<br>**AND/OR**<br>Narcotic analgesia required for symptomatic improvement	NA
Neuro-motor	Mild weakness resulting in minimal or no interference with ADL	Moderate weakness resulting in greater than minimal interference with ADL	Significant incapacity*	Paralysis<br>**AND/OR**<br>Respiratory muscle weakness resulting in ventilator dependence
Neuro-sensory	Mild impairment (decreased sensation) resulting in minimal or no interference with ADL	Moderate impairment resulting in greater than minimal interference with ADL	Significant incapacity*	NA
Neuro-cerebellar	Slight incoordination<br>**OR**<br>Dysdiadochokinesia	Intention tremor<br>**OR**<br>Slurred speech<br>**OR**<br>Nystagmus	Ataxia requiring assistance to walk or arm incoordination interfering with ADL	Unable to stand
**RESPIRATORY**				
Cough (for aerosol studies only)	Transient resulting in minimal or no interference with ADL	Treatment-associated cough resulting in greater than minimal interference with ADL	Uncontrolled cough causing significant incapacity*	NA
Bronchospasm Acute	Transient; no Rx; FEV1 or peak flow reduced to 70 - 80%	Rx required; normalizes with bronchodilator; FEV1 or peak flow 50 - 69%	No normalization with bronchodilator; FEV1 or peak flow < 49%; retractions	Cyanosis or other symptoms requiring intubation and ICU hospitalisation
Dyspnoea	Dyspnoea on exertion (such as stairs)	Dyspnoea with normal activity (such as walking)	Dyspnoea at rest	Dyspnoea requiring oxygen therapy

	SEVERITY GRADING			
**PARAMETER**	**MILD**	**MODERATE**	**SEVERE**	**VERY SEVERE**
**MISCELLANEOUS**				
Arthritis	NA	Any pain with inflammation, erythema, or joint swelling that interferes with ADL	Severe pain with inflammation, erythema, or joint swelling causing significant incapacity*	Associated with clinical diagnosis of a systemic autoimmune disease
Eye	Symptoms resulting in minimal or no interference with ADL	Notable symptoms resulting in greater than minimal interference with ADL	Symptoms (such as loss of vision, clinically diagnosed uveitis, or glaucoma) resulting in significant incapacity*	NA
Skin (general)	Scattered macular or popular eruption<br>OR<br>Erythema that is asymptomatic	Scattered macular<br>OR<br>Papular eruption<br>OR<br>Erythema with pruritis or other associated symptoms	Generalized symptomatic macular, popular, or vesicular eruption	Exfoliative dermatitis<br>OR<br>Ulcerating dermatitis
Clinical adverse events	Awareness of sign or symptom but tolerated with minimal or no interference with ADL	Notable symptoms resulting in greater than minimal interference with ADL	Symptoms causing significant incapacity*	Significant intervention/ therapy required; hospitalisation required to prevent permanent impairment or death

5. ADDITIONAL LABORATORY EVENTS				
**HEMATOLOGY**				
Fibrinogen—Elevated	450 – 600 mg/dL	601 – 650 mg/dL	> 650 mg/dL	--------------
Fibrinogen-Decreased	100 – 200 mg/dL	< 100 mg/dL	< 75 mg/dL	< 50 mg/dL **OR** associated with gross bleeding **OR** associated with disseminated coagulation
Prothrombin Time (PT)	1.1 – 1.24 x ULN	1.25 – 1.49 x ULN	1.5 – 3.0 x ULN	> 3.0 x ULN
PTT	1.1 – 1.66 x ULN	1.67 – 2.33 x ULN	2.34 – 3.0 x ULN	> 3.0 x ULN
**CHEMISTRIES**				
BUN	25 – 30 mg/dL	31 – 40 mg/dL	41 – 50 mg/dL	> 50 mg/dL
LDH	1.5 – 2.5 x ULN	>2.5 – 3.5 x ULN	>3.5 – 5.0 x ULN	> 5.0 x ULN
SODIUM<br>Hyponatremia	130 – 135 meq/L	123 – 129 meq/L	116 – 122 meq/L	< 116 meq/L
Hypernatremia	146 – 150 meq/L	151 – 157 meq/L	158 – 165 meq/L	> 165 meq/L

UNCONTROLLED COPY

5. *ADDITIONAL LABORATORY EVENTS*

	SEVERITY GRADING			
**PARAMETER**	**MILD**	**MODERATE**	**SEVERE**	**VERY SEVERE**
POTASSIUM				
Hyperkalemia	5.0 – 5.5 meq/L	5.6 – 6.0 meq/L	6.1 – 6.5 meq/L	> 6.6 meq/L
Hypokalemia	3.2 – 3.4 meq/L	3.0 – 3.1 meq/L	2.5 – 2.9 meq/L	< 2.5 meq/L
PHOSPHATASE				
Hypophosphatemia	2.0 – 2.4 mg/dL	1.5 – 1.9 mg/dL	1.0 – 1.4 mg/dL	< 1.0 mg/dL
CALCIUM (corrected for albumin)				
Hypocalcemia	7.8 – 8.4 mg/dL	7.0 – 7.7 mg/dL	6.1 – 6.9 mg/dL	< 6.1 mg/dL
Hypercalcemia	10.6 – 11.5 mg/dL	11.6 – 12.5 mg/dL	12.6 – 13.5 mg/dL	> 13.5 mg/dL
MAGNESIUM				
Hypomagnesaemia	1.2 – 1.4 meq/L	0.9 – 1.1 meq/L	0.6 – 0.8 meq/L	< 0.6 meq/L
GLUCOSE<br>Hypoglycaemia	3.1 - 3.6 mmol/l<br>55 – 64 mg/dL	2.2 - 3.0 mmol/l<br>40 – 54 mg/dL	1.7 - 2.1 mmol/l<br>30 – 39 mg/dL	< 1.7 mmol/l or mental status change or coma<br>< 30 mg/dL
Hyperglycaemia (nonfasting and no prior diabetes)	6.5 – 9.0 mmol/l<br>116 – 160 mg/dL	9.1 – 14.0 mmol/l<br>161 – 250 mg/dL	14.1 – 28.0 mmol/l<br>251 – 500 mg/dL	> 28.0 mmol/l or ketoacidosis or seizures<br>> 500 mg/dL
TRIGLYCERIDES	------------------	400 – 750 mg/dL	751 – 1200 mg/dL	> 1200 mg/dL
URIC ACID				
Hyperuricemia	7.5 – 10.0 mg/dL	10.1 – 12.0 mg/dL	12.1 – 15.0 mg/dL	> 15.0 mg/dL
LIVER FUNCTION TESTS (LFTs)				
GGT	1.25 – 2.5 x ULN	>2.5 – 5.0 x ULN	>5.0 – 10.0 x ULN	> 10.0 x ULN
Alkaline Phosphate	1.25 – 2.5 x ULN	>2.5 – 5.0 x ULN	>5.0 – 10.0 x ULN	> 10.0 x ULN
PANCREATIC ENZYMES				
Amylase	1.1 – 1.5 x ULN	>1.5 – 2.0 x ULN	>2.0 – 5.0 x ULN	> 5.0 x ULN
Pancreatic amylase	1.1 – 1.5 x ULN	>1.5 – 2.0 x ULN	>2.0 – 5.0 x ULN	> 5.0 x ULN
Lipase	1.1 – 1.5 x ULN	>1.5 – 2.0 x ULN	>2.0 – 5.0 x ULN	> 5.0 x ULN

UNCONTROLLED COPY

APPENDIX D: LABORATORY NORMAL VALUES (SITE SPECIFIC)

This appendix to be attached to this protocol by site

Test	Range	Unit
**Haematology**		
WBC		($x10^3$) cells/µL
RBC:<br>Female<br>Male		($x10^6$) cells/µL
HGB:<br>Female<br>Male		 g/dL<br>g/dL
HCT:<br>Female<br>Male		 %<br>%
PLT		($x10^3$) cells/ µL
MCV		F/L
Lymphocytes:<br>%<br>Absolute		 %<br>($x10^3$) cells/ µL
Neutrophiles:<br>%<br>Absolute		 %<br>($x10^3$) cells / µL
Monocytes: %		%
Eosinophiles: %		%
CD4:<br>%<br>Absolute		 %<br>cells / µL
CD8:<br>%<br>Absolute		 %<br>cells / µL
**Chemistry**		
ALT (SGPT):<br>Female<br>Male		IU/L
AST (SGOT):		IU/L
TOTAL BILI		µmol/L
DIRECT BILI		µmol/L
CREATININE		µmol/L

UNCONTROLLED COPY
